# Metabolically active and polyploid renal tissues rely on graded cytoprotection to drive developmental and homeostatic stress resilience

**DOI:** 10.1242/dev.197343

**Published:** 2021-04-26

**Authors:** Katie Burbridge, Jack Holcombe, Helen Weavers

**Affiliations:** School of Biochemistry, Biomedical Sciences, University of Bristol, Bristol BS8 1TD, UK

**Keywords:** Renal system, Morphogenesis, Stress resilience, Cytoprotection, DNA damage, Inflammation, Oxidative stress, Polyploidy, Mitochondria, Metabolic activity, *Drosophila*

## Abstract

Body tissues are frequently exposed to stress, from toxic byproducts generated during cellular metabolism through to infection or wounding. Although it is well-established that tissues respond to exogenous injury by rapidly upregulating cytoprotective machinery, how energetically demanding tissues – vulnerable to persistent endogenous insult – withstand stress is poorly understood. Here, we show that the cytoprotective factors Nrf2 and Gadd45 act within a specific renal cell subtype, the energetically and biosynthetically active ‘principal’ cells, to drive stress resilience during *Drosophila* renal development and homeostasis*.* Renal tubules lacking Gadd45 exhibit striking morphogenetic defects (with cell death, inflammatory infiltration and reduced ploidy) and accumulate significant DNA damage in post-embryonic life. In parallel, the transcription factor Nrf2 is active during periods of intense renal physiological activity, where it protects metabolically active renal cells from oxidative damage. Despite its constitutive nature, renal cytoprotective activity must be precisely balanced and sustained at modest sub-injury levels; indeed, further experimental elevation dramatically perturbs renal development and function. We suggest that tissues requiring long-term protection must employ restrained cytoprotective activity, whereas higher levels might only be beneficial if activated transiently pre-emptive to exogenous insult.

## Abstract (precis)

**Highlighted Article:** The cytoprotective factors Nrf2 and Gadd45 drive resilience to endogenous oxidative and genotoxic stress in metabolically-active *Drosophila* renal cells during development and homeostasis, and are further elevated following exogenous insult.

## INTRODUCTION

Throughout their lifespan, body tissues are exposed to a variety of stressors, ranging from endogenous toxic byproducts (e.g. reactive oxygen species; ROS) generated in inflammation, cellular metabolism and physiology, through to exogenous insult (e.g. UV irradiation, microbial infection or mechanical injury) (Finkel and Holbrook, 2000). These stressors can commence early during embryonic development and persist throughout an organism's life. To combat exogenous injury, tissues rapidly induce powerful pro-regenerative and cytoprotective mechanisms in response to insult, which help to protect against collateral damage and confer resilience to the passing threat (Weavers et al., 2019; Telorack et al., 2016; Medzhitov et al., 2012; Bise et al., 2019). However, some body tissues encounter significant stress during their normal development and physiology (even in the absence of exogenous challenge), suggesting that these tissues must employ robust self-defense strategies more constitutively to support tissue health and function.

One organ that is particularly vulnerable to stress-induced damage, even in the absence of exogenous insult, is the kidney. The kidney is one of the most energetically demanding organs in the human body (Bhargava and Schnellmann, 2017), consuming ∼10% of total body oxygen used in respiration (Hansell et al., 2013; Wang et al., 2010) despite its small size (∼0.5% body weight) and is therefore particularly vulnerable to oxidative stress. In fact, oxidative stress is a key factor in the development of chronic kidney disease (Daenen et al., 2019). Kidneys also perform important roles in waste detoxification and as an unavoidable consequence are exposed to large quantities of toxic byproducts (Pizzorno, 2015). Kidneys must therefore possess potent cytoprotective mechanisms to drive day-to-day resilience and prevent organ dysfunction. Although it is known that kidneys activate adaptive mechanisms in response to exogenous insult (Honda et al., 1987; Bonventre, 2002), the mechanisms by which kidneys protect themselves from persistent endogenous stress remain poorly understood. As experimentally induced constitutive cytoprotection is often harmful (Schäfer et al., 2014; Hiebert et al., 2018), it is unclear how tissues requiring long-term protection cope without suffering adverse effects. Given there is mounting interest in using cytoprotective activators therapeutically, it is crucial that we understand how these pathways function during normal tissue development and homeostasis, and how they might best be modulated for effective treatment.

*Drosophila* has recently emerged as a powerful *in vivo* model to investigate the molecular mechanisms driving stress resilience by exploiting their unrivalled opportunities for *in vivo* imaging and cell type-specific genetic manipulation (Weavers et al., 2019; Mundorf et al., 2019). Indeed, it was recently demonstrated that, upon injury, the damaged *Drosophila* epithelium rapidly and transiently upregulates a cytoprotective signaling network that protects the repairing tissue from inflammation-associated damage (Weavers et al., 2019); this network includes activation of the transcription factor Nrf2 (also known as Cnc), a ‘master regulator’ of the oxidative stress response that, in the absence of oxidative stress, is normally targeted for proteasomal degradation by its cytoplasmic inhibitor Keap1 (Suzuki and Yamamoto, 2015). Injury also induces the upregulation of *Gadd45*, the *Drosophila* homolog of the vertebrate Gadd45 gene family that is activated in response to environmental or genotoxic stress and has been implicated in a range of cellular stress responses including DNA damage repair and cell survival (Liebermann and Hoffman, 2008).

*Drosophila* possess renal (‘Malpighian’) tubules (MpTs) which play vital roles in waste excretion, osmoregulation and xenobiotic detoxification, and are well-established models to study conserved features of renal system development, function and disease (Dow and Romero, 2010; Cohen et al., 2020; Denholm, 2013). Strikingly, insect renal tubules are regarded as the fastest fluid-transporting epithelia known in biology (Maddrell, 1991); as such, *Drosophila* MpTs are highly metabolically active, with the main ‘principal’ cell type being immensely enriched with mitochondria to support the huge energy demands of these active cells (particularly for ATP-dependent ion transport) (Terhzaz et al., 2010a). This suggests that the renal tubules encounter an intrinsically high production of mitochondrial ROS (as an inevitable byproduct from the electron transport chain) and must possess robust cytoprotective machinery to resist oxidative stress. Indeed, microarray expression data (Chintapalli et al., 2007; Terhzaz et al., 2010b) suggest that adult MpTs are particularly enriched with antioxidant enzymes (e.g. catalase) even in the absence of environmental challenge. *Drosophila* renal tubules thus represent a valuable, underexploited system in which to investigate defense against ROS and other endogenous stressors that arise during tissue morphogenesis and homeostasis.

Here, we find that the cytoprotective genes Gadd45 and Nrf2 drive stress resilience within the metabolically and biosynthetically active renal principal cells (PCs) from early in development through to physiological maturity, even in the absence of exogenous insult. The DNA repair factor Gadd45 is required from mid-embryogenesis onwards, to ensure tubule cell survival and limit inflammatory infiltration, whereas Nrf2 is active from post-embryonic development once tubules have commenced their (energetically demanding) physiological activity. Strikingly, the spatio-temporal activity of Nrf2 within the renal tubules correlates precisely with that of increased mitochondrial activity and rapid tubule secretion, suggesting that Nrf2 mediates ROS protection during periods of intense metabolic and physiological activity. Consequently, mature tubules lacking Gadd45 or Nrf2 are more susceptible to DNA and oxidative damage, and adult hosts exhibit markedly reduced survival and dramatic edema, characteristic of defects in renal osmoregulation. Despite their constitutive expression, however, levels of resilience factors must be precisely regulated, as their persistent experimental elevation perturbs tubule development and host survival. We suggest that tissues requiring long-term developmental and homeostatic stress resilience must employ restrained cytoprotective activity and higher levels might only be beneficial if activated transiently (e.g. in response to exogenous insult). Given that Gadd45 and Nrf2 are expressed within the developing and mature murine and human kidney (Rees et al., 1999; Zhang et al., 1999; Schwab et al., 2003), we envision that insights from our work have wide-ranging clinical implications.

## RESULTS

### Cytoprotective genes are active within renal tubule cells *in vivo* during development and homeostasis

**Fig. 1.**
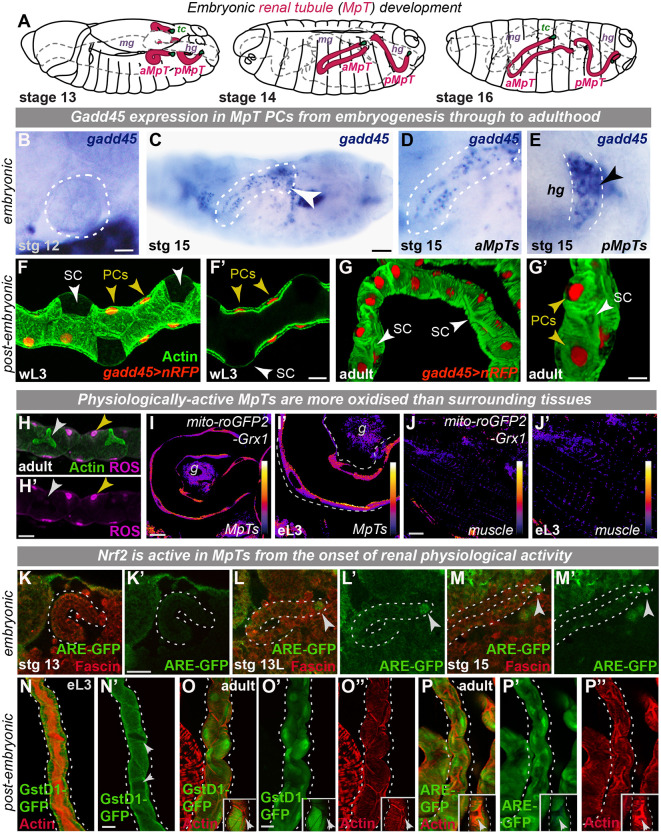
**Cytoprotective factors Nrf2 and Gadd45 are active in renal tubules *in vivo.*** (A) Schematic of *Drosophila* Malpighian tubule (MpT) embryonic development. (B-G′) Gadd45 expression within MpTs from embryogenesis (arrowheads, B-E; *in situ* hybridization) into adulthood (F-G; red, *gadd45-gal4>UAS-nRFP* and green, Phalloidin staining). White dashed lines indicate aMpT cross-section (B), lateral view of aMpT (C,D) and dorsal view of pMpTs crossing hindgut (E). (H-J′) Reactive oxygen species (ROS) levels within MpTs (DHE stains superoxide, H; PC, yellow arrowhead; SC, white arrowhead) and ratiometric *mito-roGFP2-Grx1* indicates oxidized mitochondrial glutathione (I-J′). (K-P″) Nrf2 activity in embryonic (K-M; tip cells, arrowheads) and post-embryonic (N-P; SCs, arrowheads in insets) MpTs (green, ARE-GFP or GstD1-GFP transgenic reporter; red, Phalloidin staining). White dashed lines indicate outline of renal tubules. All images representative of >10 embryos or >10 tubules from independent hosts examined per developmental stage per experiment. aMpT, anterior Malpighian tubules; eL3, early 3rd instar larva; g, gut; hg, hindgut; L2, 2nd instar larva; L3, 3rd instar larva; mg, midgut; PC, principal cell; pMpT, posterior Malpighian tubules; SC, stellate cell; tc, tip cells; wL3, wandering 3rd instar larva. See also Fig. S1 and Movie 1. Scale bars: 5 µm (B); 20 µm (C,F-P).

We sought to investigate whether cytoprotective machinery (such as that induced by skin wounding; Weavers et al., 2019) is required within vulnerable tissues to tolerate endogenous stress during development and homeostasis. *Drosophila* MpTs consist of two pairs of blind-ending epithelial tubes (Jung et al., 2005) that evert from the embryonic hindgut and proliferate to reach their mature cell number by stage 13 (Fig. 1A). MpT development is subsequently post-mitotic and MpTs undergo highly stereotypic cell rearrangements (Denholm, 2013), with convergent extension movements increasing tubule length and concomitant navigation to reach specific 3D positions by stage 16 (Fig. 1A; Movie 1). Renal tubules then persist through subsequent larval stages, metamorphosis and into adulthood. Tubules consist of two major cell types, the metabolically active PCs that support active cation transport and the smaller stellate cells (SCs) that express aquaporin water channels and control chloride flux (Dow, 2009; Cabrero et al., 2020). Strikingly, we found that the DNA repair factor Gadd45 is expressed strongly in the PCs of renal tubules from late embryogenesis (Fig. 1B-E; Fig. S1A,B) and expression is maintained throughout larval stages (Fig. 1F; Fig. S1C,D) and into adulthood (Fig. 1G; Fig. S1E,F), with minimal expression observed in SCs (arrows, Fig. 1F,G).

Physiologically active renal tubules will encounter a range of stressors and could employ multiple complementary resilience strategies for fuller protection. Indeed, renal tubules are highly metabolically active to support rapid ATP-dependent ion transport and fluid secretion and are likely to experience significant oxidative stress from mitochondrial ROS generated as a metabolic byproduct (Shadel and Horvath, 2015). *In vivo* ROS detection indicated that post-embryonic MpT PCs generate superoxide (Fig. 1H) and possess more oxidised mitochondrial glutathione (Fig. 1I,J) and higher mitochondrial H_2_O_2_ (Fig. S1G,H) compared with surrounding tissues (Albrecht et al., 2011). Consistent with this, we found that the *Drosophila* homolog (*cap'n’collar*, *cncC*) (Sykiotis and Bohmann, 2008) of the vertebrate transcription factor Nrf2, a master regulator of the oxidative and electrophilic stress response (Wakabayashi et al., 2010), is active within MpT cells (Fig. 1K-P, Fig. S1I) using *in vivo* reporters of Nrf2 activity (ARE-GFP) and Nrf2 target expression (GstD1-GFP).

Although Nrf2 is expressed ubiquitously at a low level during embryonic development (McGinnis et al., 1998), Nrf2 activity was largely absent from embryonic MpTs (Fig. 1K-M). Indeed, analysis of Nrf2 protein localization (using GFP-tagged Nrf2) demonstrated that only minimal levels of Nrf2 were observed within MpTs during embryonic development and these are predominantly cytoplasmic (Fig. S1J). Given that Nrf2 can be controlled at the post-translational level by Keap1-mediated proteasomal degradation (Suzuki and Yamamoto, 2015), we envisioned that in the absence of redox stress during embryonic development, Nrf2 protein could be degraded. However, robust Nrf2 activity was detected in MpTs from the onset of MpT physiological activity early in larval development (Fig. 1N; Fig. S1I) through to adulthood (Fig. 1O,P). Consistent with this, Nrf2-GFP was also observed within renal tubule PC nuclei from the very end of embryogenesis onwards into adult life (Fig. S1K-N). Intriguingly, the onset of Gadd45 expression and Nrf2 activity occurs at different times during renal development, which might reflect their different roles in protecting renal tubules against stress.

### Spatio-temporal Nrf2 activity correlates with renal metabolic activity and tubule physiology

**Fig. 2.**
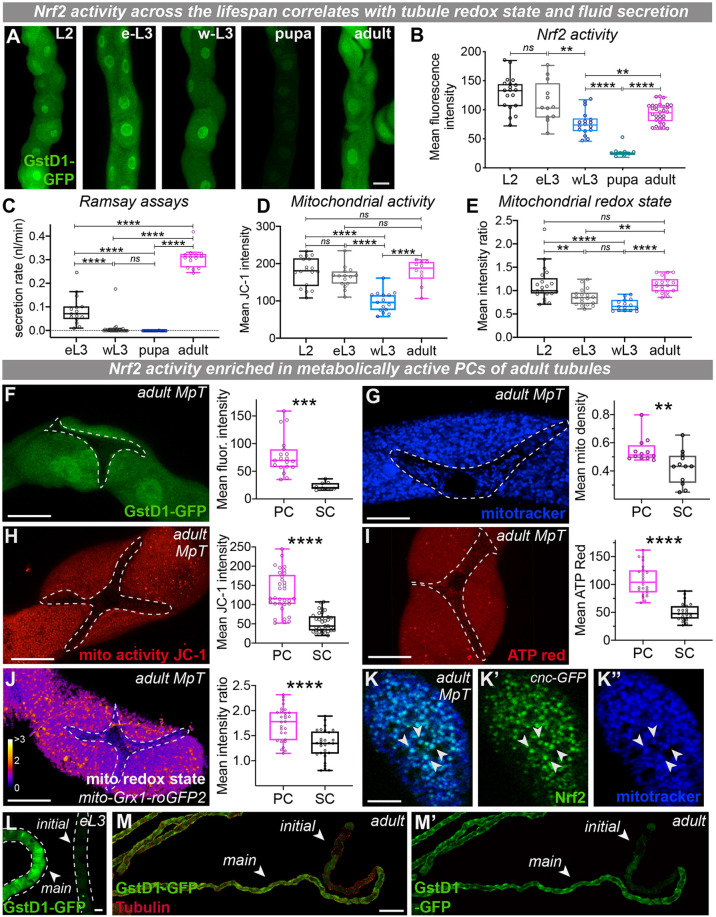
**Spatio-temporal dynamics of Nrf2 activity correlates with renal tubule metabolic and physiological activity.** (A-E) Analysis and quantification of renal tubule Nrf2 activity (A,B; green, GstD1-GFP), secretion rate (Ramsay assays, C), mitochondrial activity (JC-1 staining, D) and relative mitochondrial redox state (E, *mito-roGFP2-Grx1*; quantification performed on apical *z*-sections) across developmental stages into adulthood (in MpT main segment). (F-J) Nrf2 activity (F; green, GstD1-GFP), mitochondrial density (G; blue, MitoTracker), mitochondrial activity (H; red, JC-1), bioenergetic output (I; red, ATP-Red) and relative mitochondrial redox state (J; *mito-roGFP2-Grx1*) in principal cells (PCs) and stellate cells (SCs) (indicated by dashed outlines) in adult MpTs. (K-K″) Nrf2 co-localizes with mitochondria in PCs (arrowheads, K-K″; green, *cnc-GFP*; blue, MitoTracker). (L-M′) Nrf2 activity along the proximal-distal axis of adult MpTs (green, GstD1-GFP and red, Tubulin; 3rd instar larvae, L and adults, M). Main, main segment; initial, initial (distal) segment of renal tubule. Data represented as box and whisker plots (box, 25th to 75th percentiles; line, median; whiskers, minimum and maximum values) with all data from MpT sections (B-E) or cells (F-J) shown as overlaid points. **P<0.01, ***P<0.001, ****P<0.0001 [one-way ANOVA with multiple comparisons (B-D) or unpaired two-tailed *t*-tests (F-J)]. For JC-1 and ratiometric analysis, three images of different sections of the MpT per fly were imaged. All images representative of >10 tubules per developmental stage (B,L,M); 15 eL3, 26 wL3, 27 72 h APF pupa and 14 7-day adult MpTs (C); 6 L2, 5 eL3, 5 wL3 and 4 7-day adults (D); 7 L2, 6 eL3, 5 wL3 and 6 7-day adults (E); tubules from 5-8 flies, 12-33 cells (F-J). APF, after puparium formation; eL3, early 3rd instar larva; L2, 2nd instar larva; MpT, Malpighian tubule; ns, not significant; wL3, wandering 3rd instar larva. See also Fig. S2. Scale bars: 20 µm (A,F-L); 100 µm (M).

Strikingly, the precise spatial and temporal pattern of Nrf2 activity correlated with that of renal tubule metabolic and physiological activity (Fig. 2; Fig. S2). A comprehensive analysis of Nrf2 activity across tubule development revealed that, although Nrf2 activity was particularly high in larval MpTs, renal Nrf2 activity declined towards the end of larval life (in wandering L3 and during pupation) but significantly increased again in adulthood (Fig. 2A,B). It has been proposed that renal tubules sustain high metabolic (mitochondrial) activity to support ATP-dependent active transport (and tubule secretion) across the tubular epithelium during larval and adult life, but this temporarily shuts-down during metamorphosis (Cohen et al., 2020; Wessing and Eichelberg, 1978). Indeed, we found that larval (early L3) and adult tubules exhibited rapid tubule secretion rates (Ramsay assays, Fig. 2C) and this was associated with renal tubule cells possessing a highly active, hyperpolarized mitochondria (high membrane potential shown via JC-1 dye aggregation, Fig. 2D). These periods of increased mitochondrial activity and rapid tubule secretion also correlated with a relative shift towards a more oxidised mitochondrial redox state, indicative of increased mitochondrial ROS production (oxidised mitochondrial glutathione, Fig. 2E; Fig. S2A). MpT secretion rates dramatically declined during metamorphosis (in wandering L3 and in pupal life, Fig. 2C) and this was associated with significantly reduced mitochondrial membrane potential (Fig. 2D) and mitochondrial ROS (Fig. 2E). We thus suggest that Nrf2 is activated within the tubules during periods of intense mitochondrial ATP production (that supports tubule secretion) to protect tubules against oxidative stress.

Within physiologically active MpTs, Nrf2 activity is also highly cell-type specific – being predominantly active in PCs of adult MpTs (Fig. 2F; Fig. S2B,C) with significantly lower levels detected in SCs – which we suggest reflects the intense energetic demands of PCs that makes them more vulnerable to metabolically derived oxidative stress. Indeed, the highly metabolically active PCs possessed a richer (Fig. 2G; Fig. S2D) and more active mitochondrial complement (Fig. 2H) than their less metabolically active SC counterparts. This apparent elevation of mitochondrial activity in PCs was also highlighted by their greater bioenergetic output (as indicated by ATP production, Fig. 2I) which is likely required to support the actively maintained proton gradient for tubule secretion. Consistent with elevated mitochondrial activity driving cellular ROS production, we found that PCs possessed a significantly more oxidised mitochondrial redox state than SCs (Fig. 2J). These data suggest that Nrf2 is required in PCs to protect them from mitochondrial activity-induced oxidative stress. Intriguingly, Nrf2 itself appeared to colocalize with mitochondria in PCs (Fig. 2K), suggesting it could act as a mitochondrial stress sensor (see Discussion).

In addition to being cell-type specific, we found that Nrf2 activity was also particularly enriched within PCs in specific MpT domains, with strongest activity in the main segment (Fig. 2L,M). This pattern closely mirrors the spatial arrangement of secretory activity along the tubule proximo-distal axis, with the main segment being highly secretory but the initial segment much less so (Dow et al., 1994; O'Donnell and Maddrell, 1995). Indeed, it also correlates with the expression profile of the V-ATPase subunits which permit active cation transport in PCs of the main segment (Sözen et al., 1997), suggesting that Nrf2 might confer ROS protection in regions along the tubule with highest metabolic activity.

### Gadd45 promotes polyploidy and cell survival during renal morphogenesis

**Fig. 3.**
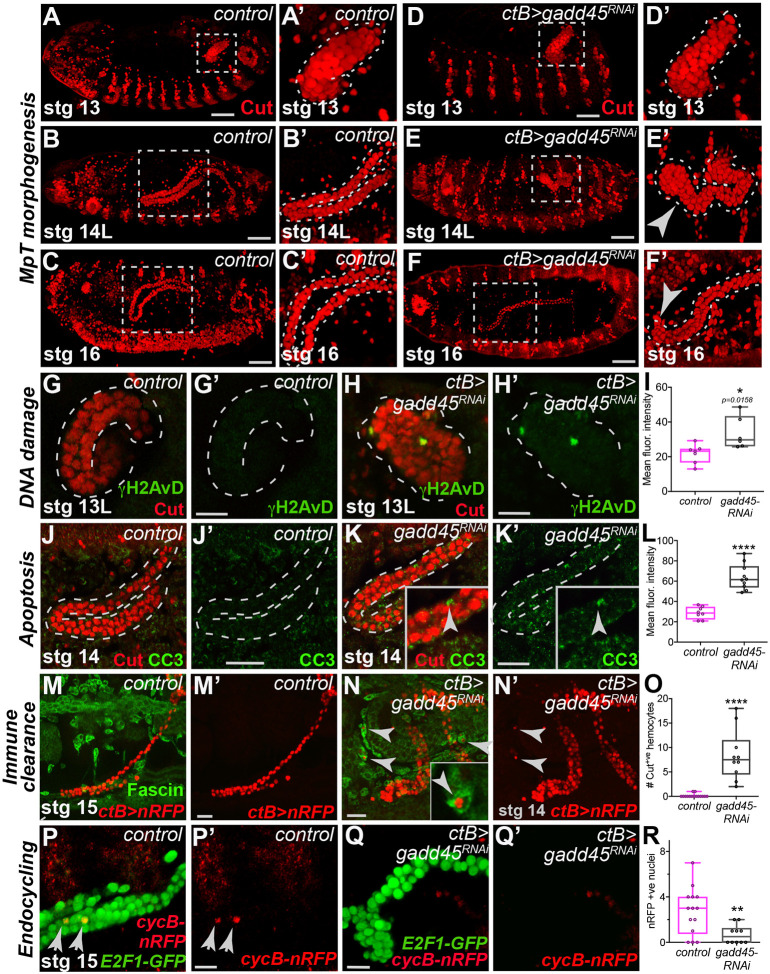
**Gadd45 limits cell death and inflammatory infiltration during renal tubule morphogenesis.** (A-F′) Malpighian tubule (MpT; red, Cut) morphogenesis in control (A-C) and *ctB>gadd45-RNAi* embryos (arrows, D-F). (G-L) DNA damage (green, γH2AvD) and apoptosis (green, CC3) in control (G,J) and *ctB>gadd45-RNAi* (H,K) MpTs. Quantifications in I and L. (M-R) *Drosophila* hemocytes (green, Fascin) in control (M) and *ctB>gadd45-RNAi* embryos (N; Cut-positive phagosomal corpses, arrowheads in insets). Quantified in O. Endocycling in control (P, Fucci labeling; red, *cyclin-B-RFP*) and *ctB>gadd45-RNAi* (Q) MpT cells. Quantified in R. Data represented as box and whisker plots (box, 25th to 75th percentiles; line, median; whiskers, minimum and maximum values) with all data shown as overlaid points. *P<0.05, **P<0.01, ****P<0.0001 (unpaired two-tailed *t*-tests). ns, not significant. All images representative of >15 embryos (A-F), >7 embryos (G-I), >8 embryos (J-L) and >10 embryos (M-R) examined per genotype, condition or developmental stage. See also Fig. S3 and Movies 2,3. Scale bars: 40 µm (A-F); 20 µm (G-Q).

We next explored whether the cytoprotective gene Gadd45 is required during MpT development and homeostasis. Although Gadd45 genes have been implicated in promoting DNA damage repair and cell survival in response to genotoxic insult e.g. irradiation (Liebermann and Hoffman, 2008), their physiological roles *in vivo*, particularly during tissue development, remain relatively unexplored. In our study, RNAi-mediated knockdown of Gadd45 within embryonic MpT PCs (using *ctB-Gal4*) resulted in marked defects in MpT morphogenesis (Fig. 3); unlike wild-type tubules that adopted precise shapes and positions in the body cavity (Fig. 3A-C), tubules lacking Gadd45 exhibited dramatic defects in this process, failing to elongate throughout their length and reach stereotypical 3D positions (Fig. 3D-F; Fig. S3A,B). However, SCs were present even in the absence of Gadd45 (Fig. S3C). Similar MpT defects were observed using an alternative early embryonic tubule driver (*byn-Gal4*) (Kispert et al., 1994; Denholm et al., 2003) or amorphic *gadd45^F17^* mutants (Fig. S3D-F).

To investigate the molecular mechanism(s) responsible for these dramatic morphological defects, we first examined DNA damage (via γH2AvD) and apoptosis (via cleaved caspase-3; CC3). Although γH2AvD and CC3 were absent from control tubules (Fig. 3G,J), strong γH2AvD signals were present in a number of *gadd45-RNAi* tubule cells (Fig. 3H,I) and some even appeared apoptotic (Fig. 3K,L), suggesting that some MpT cells might become irreversibly damaged in the absence of Gadd45. We speculated further that MpT corpses could be phagocytosed and cleared by the innate immune system (*Drosophila* hemocytes). Although hemocytes are required for normal tubule guidance (Bunt et al., 2010), hemocytes associated with control tubules very rarely contained tubule-derived debris (Fig. 3M; Fig. S3G). In contrast, numerous hemocytes associated with *gadd45-RNAi* MpTs contained Cut-positive corpses in their phagosomes (Fig. 3N,O; Fig. S3H), consistent with our live-imaging data that *gadd45-RNAi* tubule cells (with condensed nuclei) often exited the developing tubules (Movies 2 and 3). This phenotype mirrors that reported in *GADD45a* KO mice, which develop chronic nephropathy with glomerular inflammation (Salvador et al., 2002). As inflammatory cells that have been locally activated could generate inflammatory ROS (Nguyen et al., 2017), this could set up a self-amplifying cycle of oxidative stress, tissue damage and inflammation in the renal microenvironment; by limiting the amount of cell death, we envision that cytoprotection acts as an important barrier to tissue inflammation.

From stage 13, MpT PCs are post-mitotic and replicate DNA via endocycling (Skaer, 1989), a process often used to expand the genome of biosynthetically active cells (Lilly and Duronio, 2005; Gandarillas et al., 2018). However, DNA lesions can arise during DNA replication and must be repaired for continued cycling; perhaps Gadd45 functions during rapid MpT endocycling to maintain genome integrity and cell survival? Unlike control MpT PCs that increased ploidy from 2C to 4-8C by stage 16 (Fig. S3I-K), the ploidy of *gadd45-RNAi* MpT PCs remained lower, suggesting they progressed through fewer endocycles (Fig. S3L,M). This was supported by Fucci cell cycle labeling (Zielke et al., 2014), as control cells moved through S-phase (with *cyclinB-RFP*) (Fig. 3P; Fig. S3N-P) but this was rarely observed in *gadd45-RNAi* MpT cells, which remained in G1 (*E2F1-GFP*; Fig. 3Q,R; Fig. S3Q,R). These data suggest that Gadd45 is pivotal for stress resilience within endocycling tubule cells during renal morphogenesis, to limit DNA damage, apoptosis and inflammatory infiltration, and ultimately to ensure renal tubules adopt their stereotypical 3D architecture.

### Gadd45 confers stress resilience in mature polyploid renal tubules

**Fig. 4.**
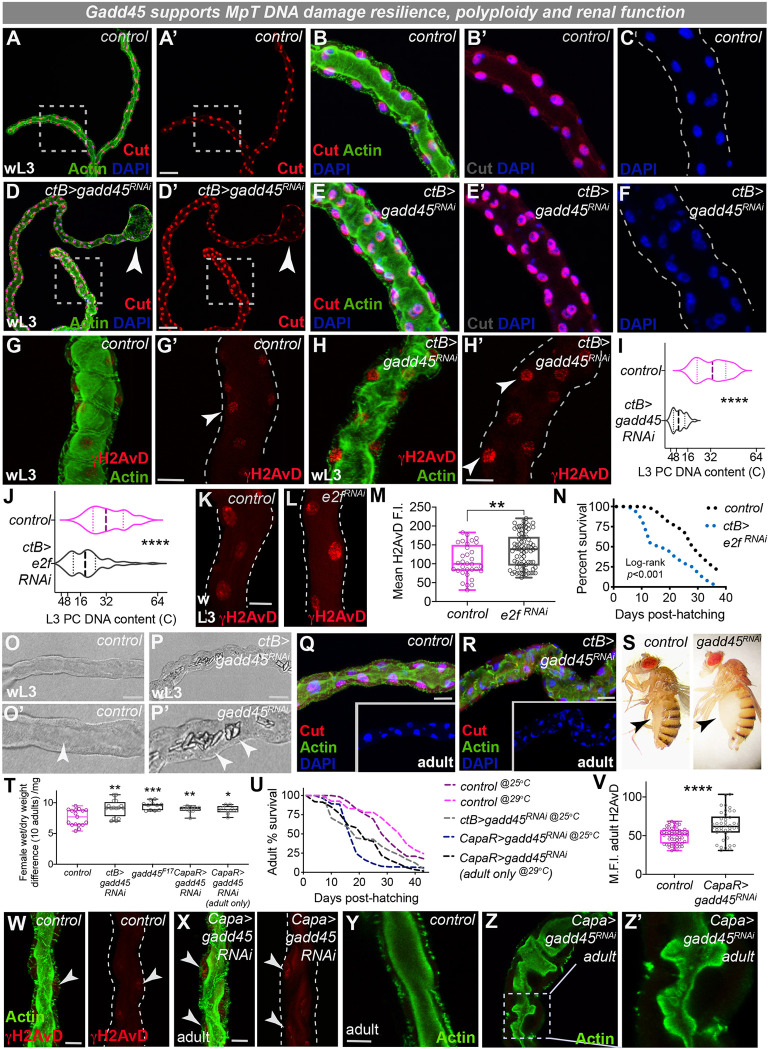
**Gadd45 drives stress resilience within endocycling renal cells to support renal function and host survival.** (A-F) Larval Malpighian tubule (MpT) morphology (red, Cut; green, Actin; blue, DAPI) in control (A-C) and *ctB>gadd45-RNAi* (D-F). Arrowheads indicate dilated tips. B-C and E-F show magnification of boxed areas in A and D, respectively. (G-I) Analysis of DNA damage (red, γH2AvD; arrowheads, G-H′) and ploidy (I). (J-N) Inhibition of PC endocycling via *ctB>e2f-RNAi*-reduced PC ploidy (J), elevated DNA damage signaling (K,L; red, γH2AvD), quantified in M, and reduced adult survival (N; log-rank *P*<0.001). (O-U) Luminal crystals (arrowheads, O-P′), abnormal morphology (Q,R), abdominal bloating (S,T) and reduced lifespan (U, *n*>50 per group) in control and *gadd45-RNAi* animals. (V-Z′) DNA damage (V,W; red, γH2AvD) and tubule morphology (Y,Z; green, Actin) were also disturbed following post-embryonic inhibition of Gadd45 using *capaR-gal4.* Log-rank comparison of survival curves (U) give test-statistics of *P*=0.0035 (control versus *ctB>gadd45-RNAi*), *P*<0.001 (control versus *capaR>gadd45-RNAi*) and *P*<0.001 (control versus *capaR>gadd45-RNAi* adult only). Data represented as violin plots (I,J; plot, frequency distribution; dashed line, median; dotted lines, interquartiles), box and whisker plots (box, 25th and 75th percentiles; line, median; whiskers, minimum and maximum values) with all data shown as overlaid points (M,T,V) or line graphs (N,U). *P<0.05, **P<0.01, ***P<0.001, ****P<0.0001 [unpaired xxx *t*-tests (I,V) or Log-rank survival analyses (N,U)]. Images representative of >10 tubules (A-F), >12 tubules (G-I and O-R), >30 PCs from >6 tubules (J-M and V-X) and >100 adults (T,U) examined per genotype, condition or developmental stage. Dashed lines (G′,H′,K,L,W,X) indicate renal tubule outlines. wL3, wandering 3rd instar larva. See also Fig. S4. Scale bars: 40 µm (A,D,O-P); 20 µm (G-H′,K,L,Q,R,W-Y).

We next examined how Gadd45-mediated cytoprotection impacts on renal tissue once the tubules reach physiological maturity. The morphological defects of *gadd45-RNAi* MpTs persisted into post-embryonic life (Fig. 4A-H); unlike the long thin control tubules (Fig. 4A-C; Fig. S4A), *gadd45-RNAi* tubules were abnormal in shape (with more PCs around the lumen) (Fig. 4D-F; Fig. S4B) and sometimes possessed dramatically dilated distal tips (Fig. 4D). Although SCs were present within *gadd45-RNAi* tubules (small nuclei, Fig. S4C′,D′), they were irregularly spaced along the tubule length (Fig. S4D′). Similar abnormalities were observed in *gadd45^F17^* mutants (Fig. S4E,F). Moreover, larval MpT cells lacking Gadd45 possessed significantly more DNA damage (Fig. 4H; Fig. S4G) than controls (Fig. 4G), and oxidative damage (8-oxodG staining) was also significantly affected (Fig. S4H).

Given that unrepaired DNA damage could block DNA replication, we also explored tubule cell ploidy. Endocycling continued in post-embryonic MpTs and the DNA content increased to 64C by the 3rd larval instar (Fig. 4I; Fig. S4I). However, in *gadd45-RNAi* tubules, PC DNA content remained at 4-16C, suggesting that *gadd45-RNAi* cells endocycle far less than normal (Fig. 4I; Fig. S4I,J). The nuclei of *gadd45-RNAi* PCs were also significantly smaller than controls (perhaps reflecting lower DNA content, Fig. S4K) and many lacked Fucci staining (Fig. S4M), suggesting that they may have entered an inactive resting state (unlike control PCs, Fig. S4L). By promoting the repair of DNA damage (perhaps via interactions with PCNA; Liebermann and Hoffman, 2008), Gadd45 could thus ensure normal endocycling to generate polyploid mature tubule cells.

Intriguingly, recent studies have linked polyploidy to increased stress resistance (Herrtwich et al., 2016; Grendler et al., 2019). We therefore tested the effect of inhibiting PC endoreplication during tubule development (Fig. 4J-N) by RNAi-mediated inhibition of the cell cycle transcription factor E2f1, a strategy used previously to inhibit endoreplication in *Drosophila* (Losick et al., 2013); although *ctBGal4*-driven expression of *e2f-RNAi* did not adversely affect embryonic tubule morphogenesis (Fig. S4N,O), tubule cells had lower ploidy than controls (Fig. 4J; Fig. S4P) and accumulated increased levels of DNA damage-associated γH2AvD staining (Fig. 4K-M; Fig. S4Q). Moreover, adult flies exhibited shorter lifespans (Fig. 4N) and experienced more edema than controls (with female wet/dry weight differences mean=9.9 mg per 10 adults versus control mean=7.5 mg per 10 adults, *P=*0.0274, unpaired *t*-test), suggesting that increased tubule ploidy confers beneficial effects at both a cellular and whole organism level. Given that many cells within the vertebrate kidney also endocycle (Duncan, 2013; Wang et al., 2018), polyploidy may be a conserved mechanism for promoting stress resilience in vulnerable tissues.

The mature shape of renal systems is crucial for their effective physiological function in both flies and mammals (Saxena et al., 2014; Mulroy et al., 2003). Indeed, we found that loss of renal Gadd45 was associated with striking adult physiological defects and host lethality (Fig. 4O-Z). To separate out the requirement for Gadd45 during early embryonic development and later post-embryonic life, we assessed adult tubules from hosts in which Gadd45 expression had been inhibited from embryonic tubule development (using *ctB-gal4*-driven RNAi or *gadd45^F17^* amorphic mutants), as well as those in which Gadd45 was inhibited during post-embryonic life only (using *capaR-Gal4*; Terhzaz et al., 2012). MpTs lacking Gadd45 throughout development (*ctB>gadd45-RNAi)* accumulated large luminal polyangular birefringent concretions (resembling calcium oxalate stones; Chen et al., 2011; Teigler and Arnott, 1972) (Fig. 4O,P) and adult flies with malformed MpTs (Fig. 4Q-R) displayed abdominal bloating (edema) (Fig. 4S; Fig. S4R) and fluid retention (Fig. 4T), indicative of defective osmoregulation (Saxena et al., 2014). These tubule defects were associated with poor host survival rates (Fig. 4S,T) and significantly shorter lifespans than controls (Fig. 4U; Fig. S4U). Similar phenotypes were observed in tubules in which Gadd45 was inhibited from late larval life (*CapaR>gadd45-RNAi*), which exhibited increased DNA damage (Fig. 4V-X), reduced survival (Fig. 4U) and bloating (Fig. 4T). To more specifically test the role of Gadd45 within adult MpTs, we employed a temperature-sensitive Gal80 construct in combination with *CapaR-Gal4* and *UAS-gadd45-RNAi* to knock down *gadd45* only in adult PCs; we found that these adult flies also exhibit reduced survival compared with control flies raised in identical conditions (Fig. 4U), as well as increased bloating (Fig. 4T), and that the tubules themselves had morphological defects with abnormal luminal actin (Fig. 4Y,Z). Strikingly, the most severe survival phenotypes were observed in *gadd45^F17^* null mutants that lacked Gadd45 throughout development (Fig. S4U), suggesting that Gadd45 is required both early during tubule morphogenesis as well as in post-embryonic life to sustain tubule cell health and host fitness.

### Nrf2 drives oxidative stress resilience in physiologically active renal tubules

**Fig. 5.**
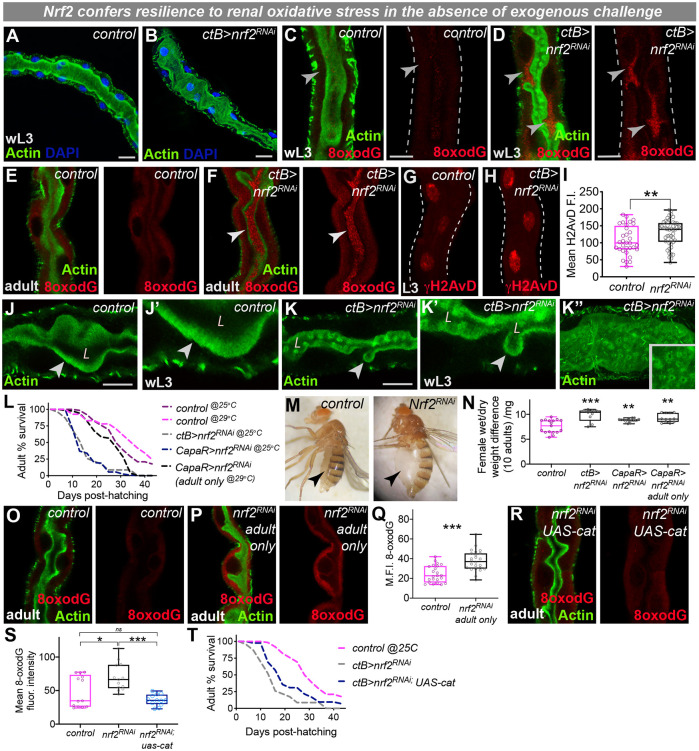
**Nrf2 confers oxidative stress protection in physiologically active renal tubules.** (A-K″) Malpighian tubule (MpT) gross morphology (A,B, L3 tubules; green, Actin; blue, DAPI), oxidative DNA damage (red, 8-oxodG; C,D, L3 tubules; E,F, adult tubules), DNA damage (red, γH2AvD; G-H; quantified in I, L3 tubules) and luminal morphology (green, Actin; J-K, L3 tubules) following *ctB-gal4* driven *nrf2-RNAi*. (L-N) Adult lifespan (L, *n*>50 per group) and edema (M,N) following *nrf2-RNAi*-driven inhibition using *ctB-gal4* or *capaR-gal4*. Log-rank comparison of survival curves (L) gave test-statistics of *P*<0.001 for control^25°C^ versus *ctB>nrf2-RNAi*, control^25°C^ versus *capaR>nrf2-RNAi* and control^29°C^ versus *capaR>nrf2-RNAi* adult only. (O-Q) Oxidative DNA damage (red, 8-oxodG) following *nrf2-RNAi* in adults only (using *capaR-gal4* and *tubGal80^ts^*). (R-T) Ectopic *catalase* expression in *ctB>nrf2-RNAi* tubules reduced oxidative damage (red, 8-oxodG; R,S) and improved adult survival (T; log-rank test statistic of *P*=0.018 for *ctB>nrf2-RNAi* versus *ctB>nrf2-RNAi;UAS-cat*). Data represented as box and whisker plots (box, 25th to 75th percentile; line, median; whiskers, minimum and maximum values) with all data shown as overlaid points, or line graphs. *P<0.05, **P<0.01, ***P<0.001 [two-tailed unpaired *t*-tests (I,Q), one-way ANOVA with multiple comparisons (N,S) or Log-rank survival analyses (L,T)]. All images representative of >7 tubules (A-D,J-K), >10 tubules (E-F,R-T), >30 PCs from >8 tubules (G-I), >100 adults (L) and >15 tubules (O-Q) examined per genotype, condition or developmental stage. M.F.I., mean fluorescent intensity; ns, not significant; wL3, wandering 3rd instar larva. See also Fig. S5. Scale bars: 40 µm (A,D,J,K); 20 µm (G,H,O,U).

We suggest that MpTs might employ a number of complementary cytoprotective strategies; indeed, Nrf2 is active within PCs during intense tubule physiological activity, suggesting that Nrf2 might confer protection from excessive mitochondrial ROS production during both larval and adult life. Consistent with the lack of embryonic Nrf2 activity, knockdown of renal Nrf2 during embryogenesis did not perturb MpT development (Fig. S5A,B), mirroring the lack of gross morphological defects in the embryonic kidneys of Nrf2 knockout mice (Chan et al., 1996). In contrast, RNAi-mediated loss of Nrf2 from post-embryonic MpTs was associated with disturbed tubule morphology (Fig. 5A-D; Fig. S5C,D) and increased oxidative damage (Fig. 5C-F; Fig. S5E-G) in larval and adult tubules, as well as increased DNA damage (Fig. 5G-I). Strikingly, loss of Nrf2 caused a dramatic increase in the level of luminal 8-oxodG (Fig. 5E,F); given that the presence of elevated urinary 8-oxodG is routinely employed as a biomarker for oxidative stress (and can predict acute renal damage) in the clinic (Shah et al., 2007; Schupp et al., 2016; Chien et al., 2014), our data could suggest that loss of Nrf2 causes damaged renal cells to release oxidised nucleosides into the tubule lumen. Tubules lacking Nrf2 also exhibited conspicuous luminal abnormalities (lacking the thick region of apical actin fluorescence; Fig. 5J,K; Fig. S5H,I), suggesting a potential defect in the microvilli normally present at the luminal surface (Halberg et al., 2016) in both larval and adult MpTs. Intriguingly, an early manifestation of mammalian acute kidney injury is the disruption of microvilli in the proximal tubule (Sharfuddin and Molitoris, 2011).

These renal defects were associated with a marked reduction in host survival (Fig. 5L) and renal physiology, with adult flies exhibiting grossly distended abdomens (Fig. 5M) and higher fluid retention (Fig. 5N; Fig. S5J), suggesting that osmoregulation is compromised in the absence of Nrf2-mediated oxidative stress protection. Given the lack of Nrf2 activity in embryonic tubules and the absence of defects in embryonic tubules expressing *nrf2-RNAi*, we propose that the phenotypes in *ctB>nrf2-RNAi* adults are due to the requirement for Nrf2 during intense PC metabolic activity in post-embryonic life. Indeed, we observed similar phenotypes using the *CapaR-gal4* driver to inhibit PC expression of Nrf2 from late larval life onwards (Fig. 5L,N). We also inhibited Nrf2 within adult tubules only (using Gal80-mediated temporal control, see above) and found that MpTs exhibited increased oxidative damage and shorter lifespans than controls (Fig. 5L,N-Q), although these phenotypes were less severe than those observed in tubules lacking Nrf2 throughout larval and adult life. Therefore, Nrf2 is required during both larval and adult life when PCs are highly active to drive oxidative stress resistance.

To explore whether these *nrf2-RNAi* phenotypes are caused by loss of ROS detoxification, we tested whether the expression of antioxidant enzymes within the tubules could perhaps (at least partially) rescue the *nrf2-RNAi* phenotype. Surprisingly, ectopic expression of the mitochondrial superoxide dismutase enzyme SOD2 (that converts superoxide to H_2_O_2_) further exacerbated the *nrf2-RNAi* phenotype, with elevated oxidative damage and poor adult survival (Fig. S5K-O); indeed, SODs have been shown to enhance H_2_O_2_-induced DNA damage (Midorikawa and Kawanishi, 2001), highlighting the importance of having a well-balanced antioxidant system. Conversely, ectopic expression of catalase (that converts H_2_O_2_ to H_2_O and oxygen) partially rescued the *nrf2-RNAi* phenotype with improved survival and reduced oxidative damage (Fig. 5R-T).

### Exogenous stress elevates MpT cytoprotective activity but long-term elevated Gadd45 and Nrf2 drives renal pathology

**Fig. 6.**
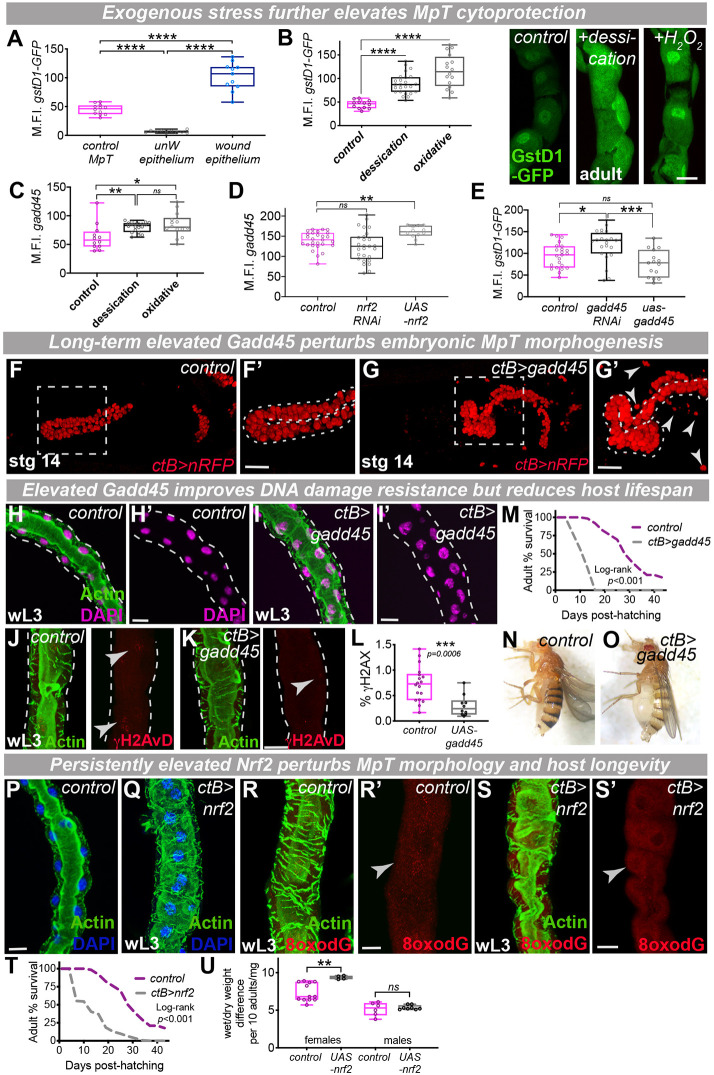
**Long-term elevated Gadd45 and Nrf2 drives renal pathology.** (A) Quantification of Nrf2 activity in adult Malpighian tubules (MpTs) (as detected using GstD1-GFP) versus unwounded epithelial tissue and repairing epithelial tissue following wounding. (B,C) Adult MpT Nrf2 activity or Gadd45 expression were elevated in response to exogenous stress (desiccation or H_2_O_2_ treatment; green, GstD1-GFP in B). (D,E) L3 MpT Gadd45 expression (D) or Nrf2 activity (E) following RNAi-mediated inhibition or UAS-mediated overexpression of Nrf2 or Gadd45, respectively. (F-O) MpT morphogenesis (red, *ctB>*nRFP; F-G, with Cut-positive nuclei outside MpTs indicated by arrowheads), larval MpT morphology (H-I; green, Actin; magenta, DAPI), DNA damage (red, γH2AvD, J-L, arrowheads indicate nuclear staining), adult survival (M; log-rank test statistic of *P*<0.001) and adult edema (N-O) in control and *ctB>gadd45* animals. F′ and G′ show magnification of boxed areas in F and G, respectively. (P-U) MpT morphology (P,Q), oxidative DNA damage (red, 8-oxodG; R-S, arrowheads indicate nuclear staining), lifespan (T, *n*>50 per group; log-rank test statistic of *P*<0.001) and water retention (U) in control and *ctB>nrf2* animals. Data represented as box and whisker plots (box, 25th and 75th percentiles; line, median; whiskers, minimum and maximum values) with all data shown as overlaid points or line graphs. *P<0.05, **P<0.01, ***P<0.001, ****P<0.0001 [two-tailed unpaired *t*-tests (L,U), one-way ANOVA with multiple comparisons (A-E) or Log-rank survival analyses (M,T)]. All images representative of >10 tubules or embryos (A-G), >15 tubules (H-L), >100 adults (M,T,U) and >7 tubules (P-S) examined per genotype or condition. Dashed lines (H-K) indicate outline of the renal tubule. ns, not significant; wL3, wandering 3rd instar larva. See also Fig. S6. Scale bars: 20 µm (B,H-I, J-K and P-S) and 40 µm (F-G).

Although the level of endogenous cytoprotective machinery (e.g. Nrf2 activity) in otherwise ‘unchallenged’ adult renal tubules is higher than that of adjacent tissues (e.g. the epithelium), it is substantially lower than that induced in response to exogenous injury (e.g. in a wounded epithelium) (Fig. 6A; Fig. S6A-C). Nevertheless, renal tubules can themselves display adaptive responses to exogenous insult, as renal Nrf2 activity (Fig. 6B; Fig. S6D-F) and Gadd45 expression (Fig. 6C) are elevated further in response to environmental stresses (such as desiccation or oxidative stress). Indeed, adult MpTs are ‘immunocompetent’ and respond to exogenous insult (e.g. infection) by releasing antimicrobial factors (Davies et al., 2012) and increasing clearance of damaging metabolites from the hemolymph (Li et al., 2020). Given their overlapping expression profiles and complementary roles in cytoprotection, we also tested whether levels of Gadd45 and Nrf2 impact on each other's expression or activity; intriguingly, experimental elevation of Nrf2 caused a significant increase in Gadd45 expression (Fig. 6D) whereas loss of *nrf2* was associated with a small (albeit non-significant) reduction in Gadd45 levels (Fig. 6D). Conversely, loss of *gadd45* elevated renal Nrf2 activity (consistent with the increased oxidative damage observed in these tubules), whereas upregulation of *gadd45* caused a small (albeit non-significant) reduction in Nrf2 activity (Fig. 6E).

Given the protective role of Gadd45 and Nrf2 during tubule development and that exogenous stress elevates their levels even further, we tested whether their experimental elevation might confer additional benefits. However, constitutive overexpression of Gadd45 caused marked defects in tubule morphogenesis (Fig. 6F,G; Fig. S6G,H), with inflammatory infiltration and clearance of tubule debris (Fig. S6I,J). These defects persisted during post-embryonic stages, in which MpTs with elevated Gadd45 were misshapen, with more PCs around the lumen and SCs irregularly spaced along the length (Fig. 6H,I; Fig. S6K,L). Although tubule nuclei with elevated Gadd45 were slightly larger than controls (Fig. S6M), their DNA content was lower (Fig. S6N), suggesting that elevated Gadd45 might impair tubule cell ability to endocycle. Moreover, despite a reduction in DNA-damage associated γH2AvD (Fig. 6J-L), flies with persistently elevated Gadd45 exhibited poor survival (Fig. S6O) with markedly reduced adult lifespan (Fig. 6M), abdominal distention (Fig. 6N,O) and increased water retention (Fig. S6P). We performed similar analysis by constitutively elevating Nrf2 levels within developing MpTs; strikingly, excessive Nrf2 expression disrupted post-embryonic tubule morphology, as the lumen became unusually enlarged (Fig. 6P,Q) and the tubules exhibited abnormal bulbous regions along their length (Fig. S6Q,R). Elevated Nrf2 even caused a small (but non-significant) rise in oxidative DNA damage within tubule cells (Fig. 6R,S; Fig. S6S). Accompanying these changes, constitutive elevation of renal Nrf2 caused a marked decline in adult lifespan (Fig. 6T) and increased water retention, indicative of osmoregulatory defects (Fig. 6U). Our results suggest that persistently elevated renal Gadd45 or Nrf2 negatively impacts on tubule morphology, physiological function and adult survival in unchallenged conditions. Precise regulation of cytoprotection within renal tubule cells is therefore essential to support renal tubule morphogenesis, tissue health and host longevity.

## DISCUSSION

Although recent work has demonstrated that mammalian tissues can induce cytoprotective strategies in response to acute exogenous insult, the role of cytoprotective factors in supporting normal tissue development and function remains comparatively unexplored, despite expression of resilience genes (including Gadd45 and Nrf2) in unwounded tissues, including the mammalian kidney (Rees et al., 1999; Zhang et al., 1999; Schwab et al., 2003). Kidneys are, in fact, exposed to multiple stressors even in the absence of exogenous insult, including oxidative stress (from ROS generated during cellular metabolism and physiology), DNA replication stress (from mitotic divisions and endocycling) and waste detoxification. Here, we show that the cytoprotective factors Nrf2 and Gadd45 are essential to drive stress resilience during *Drosophila* renal tubule morphogenesis and homeostasis.

Strikingly, we find that renal cytoprotection is cell-type and domain-specific, being active in those cells that require it the most, the energetically demanding and biosynthetically active PCs (Terhzaz et al., 2010a). PCs are highly metabolically active in order to drive active cation transport across the tubular epithelium (Terhzaz et al., 2010a) and excrete a wide range of solutes and, as such, are more vulnerable to oxidative stress than adjacent SCs. Indeed, we show that the contrast between the functional roles of PCs and SCs is reflected in their energetic demands and resultant metabolic stress. PCs possess a richer (and more active) mitochondrial complement than their less metabolically active SC counterparts, and although this is accompanied by a greater bioenergetic output (ATP generation), PCs also demonstrate a more oxidized mitochondrial redox state, likely indicating an increase in electron leakage and cellular ROS production. As a result of the shift to a more oxidised redox state, the colocalisation we observe of Nrf2 to the mitochondria may be acting to coordinate mitochondrial-associated stress signaling in these highly bioenergetically demanding cells (Dinkova-Kostova and Abramov, 2015). Moreover, Nrf2-mediated renal cytoprotection is also highest during periods when the tubules are actively secreting (in early larval life and adulthood) and in those tubule functional domains that perform the most ATP-dependent active transport. Given the detrimental effects of inappropriate cytoprotection, it appears vital that cytoprotective pathways are not only precisely controlled in a strict temporal manner but also a spatial one.

As tissues might experience several different types of cellular stress during their lifetime, it appears that vulnerable tissues employ multiple, complementary cytoprotective pathways. Indeed, alongside Nrf2-mediated oxidative stress protection, renal tubules require Gadd45-mediated protection against DNA damage to ensure cell survival and host longevity. Intriguingly, developmental Gadd45 expression was required to ensure renal tubule cells endoduplicated their genomes effectively; given that experimental inhibition of endocycling caused marked changes in DNA damage signaling and reduced host survival, we envision polyploidy could in itself be an important mechanism to drive increased stress resilience (Herrtwich et al., 2016; Grendler et al., 2019).

Vulnerable tissues such as the kidney thus employ a twofold protection strategy. To cope with stress encountered during normal morphogenesis and homeostasis, cytoprotective pathways are maintained at low (somewhat constitutive) levels to allow tissues to mount an appropriate protective response and ensure cell survival. If stress exceeds a certain threshold (e.g. following exogenous insult such as desiccation), cytoprotective pathways are rapidly induced further to offer additional protection; however, these higher levels might only be tolerated if the induction is transient. Indeed, cytoprotective factors are upregulated in vertebrate renal cells in response to exogenous insult (e.g. hyperosmolality; Chen et al., 2016; Mak and Kültz, 2004), just as we observed in *Drosophila* renal tubules, but we found that Gadd45 and Nrf2 quickly became pathological if levels were persistently elevated experimentally. This is consistent with recent studies demonstrating that hyperactivation of Nrf2 in the murine kidney promotes nephrogenic diabetes insipidus-like features (Suzuki et al., 2017) and experimental Nrf2 elevation (e.g. in epithelium or fibroblasts) reduces cell ‘fitness’ and drives senescence (Hiebert et al., 2018; Kucinski et al., 2017; Tsakiri et al., 2019). Cytoprotection is thus emerging as a ‘double-edged sword’ and must be carefully balanced to ensure optimal tissue protection. Although ectopic activation of these pathways above endogenous levels in response to exogenous injury can be protective, these higher levels might only be tolerated if the induction is transient (and cytoprotection returns to its original level once the threat has passed). Thus, only low-level graded activation is tolerated in the long-term and high-level activation is beneficial in the short-term.

Improved insight into the mechanisms driving stress resilience will help us understand the pathophysiology of many stress-related degenerative diseases (e.g. chronic kidney disease) and could help explain why some patients are more susceptible to developing these conditions due to inherited defects in resilience pathways. This innate cytoprotective machinery also has enormous clinical potential if it can be harnessed therapeutically – a fuller understanding of cytoprotective strategies will point to how they can be best exploited to improve stress resilience in the clinic, e.g. to protect patient tissues during surgery or organ transplant (Hausenloy et al., 2016). Given that transplanted kidneys from cadavers have reduced detoxification power against circulating toxins and suffer more oxidative stress than those from living donors, these might be prime tissues in which to boost resilience (Bocedi et al., 2018). However, in order to successfully employ cytoprotective activators therapeutically, it is essential to first understand how these protective pathways function during tissue development and physiology – and crucially, how their precise spatio-temporal dynamics (including the duration and level of activation) impacts on tissue health – to ensure optimal, safe dosing of cytoprotection agonists.

## MATERIALS AND METHODS

### *Drosophila* stocks and genetics

Fly stocks were maintained according to standard protocols (Greenspan, 1997). All crosses were performed at 25°C unless otherwise stated. The following *Drosophila* stocks were used: *ctB-gal4* (tubule-specific driver; Sudarsan et al., 2002), *byn-Gal4* [expresses in the embryonic hindgut/MpT primordium (Kispert et al., 1994) and persists in embryonic MpT PCs (Denholm et al., 2003)] *UAS-Fucci* (*E2F1-GFP; cyclinB-nRFP*), *tsh-lacZ*, *UAS-RedStinger* (nuclear RFP), *Ubi-p63E-Tubulin-RFP*, *mito-roGFP2-Orp1*, *mito-roGFP2-Grx1* (Albrecht et al., 2011), *gadd45-gal4* (Kyoto Stock Center), *ARE-GFP* (Chatterjee et al., 2012; 4XARE:GFP-16, Nrf2 activity reporter, gift from Ioannis Trougakos, University of Athens, Greece), *GstD-ARE:GFP* (Sykiotis and Bohmann, 2008; ARE of the gstD gene, gift from Ioannis Trougakos), *OregonR*, *cnc-eGFP* (#38631), *e2f-RNAi* (#27564 or #36126), *capaR-gal4* (gift from Julian Dow, University of Glasgow, UK), *tubGal80^ts^* (gift from Matthias Landgraf, University of Cambridge, UK), *UAS-catalase* (#24621), *UAS-sod2* (#24494), *UAS-Gadd45* (gift from Uri Abdu; Peretz et al., 2007), *UAS-dNrf2* (gift from Ioannis Trougakos; Sykiotis and Bohmann, 2008), *UAS-Gadd45-RNAi* (TRiP.HMS01436), *Gadd45^F17^* null mutant (Nelson et al., 2016) and *UAS-dNrf2-RNAi* (gift from Ioannis Trougakos; Sykiotis and Bohmann, 2008). For RNAi-mediated *gadd45* or *nrf2* knockdown within adult tubules only, *Drosophila* also carrying *capaR-gal4* and *tubGal80^ts^* constructs were raised at the permissive temperature (18°C) and then moved to the restrictive temperature (29°C) in adulthood. *Drosophila* mutants and transgenic lines were obtained from the Bloomington *Drosophila* Stock Center unless otherwise stated.

### Microscopy and wounding

For live-imaging, embryos of the appropriate developmental stage were collected from overnight apple juice plates, dechorionated in bleach for 1 min and mounted on double-sided sticky tape on glass slides in 10S Voltalef oil (VWR). Where required, wounds were induced using a nitrogen-pumped Micropoint ablation laser tuned to 435 nm (Andor Technologies) (Razzell et al., 2013). For imaging of larval and adult renal tubules, intact living animals were rapidly dissected in fresh ice-cold PBS (Sigma-Aldrich, P7059) using forceps before immediately transferring to glass slides in PBS for live-imaging or fixation with 4% paraformaldehyde (PFA) (Sigma-Aldrich, 8.18708) in PBS for 15 min. For ROS detection, dissected MpTs were incubated with DHE (Invitrogen, Molecular Probes) in PBS for 5 min in the dark at room temperature before several washes in PBS. For analysis of relative mitochondrial redox state (indicative of ROS production) using genetically encoded redox sensors (*mito-roGFP2-Grx1* or *mito-roGFP2-Orp1*) imaging was performed as previously described (Albrecht et al., 2011); briefly, the biosensors were excited by the 405 nm and 488 nm laser lines sequentially (and line by line) and emission was detected in the 500-570 nm range. Images (tif files) were processed using ImageJ as previously described (Albrecht et al., 2011), with the final ratio image created by dividing the 405 nm image by the 488 nm image and visualizing in false colors using the lookup table ‘Fire’. For imaging of mitochondrial activity, ATP production or mitochondrial density, live intact MpTs were dissected from control (*OrR*) flies in ice-cold Schneider's medium and stained with JC-1 (5 µg/ml, Abcam, ab113850), BioTracker ATP-Red (10 µM, Millipore, SCT045) or MitoTracker Far Red (100 nM, Invitrogen, M22426) for 8-10 min in warm Schneider's medium (Sigma-Aldrich, S0146) at room temperature in dark conditions; MpTs were mounted in 5 µl Schneider's medium for imaging.

Confocal imaging was performed on a Leica TCS SP8 confocal microscope. Image processing was performed using Volocity (PerkinElmer), ImageJ (National Institutes of Health; Schindelin et al., 2012), Adobe Photoshop or Adobe Illustrator software. For quantification of the percentage area of oxidative and DNA damage, all processing was performed in ImageJ; briefly, confocal images were converted to binary format and thresholded before using the Analyze/Measure tool to calculate the percentage area. For quantification of cell ploidy (DNA content), the integrated fluorescence intensity of renal tubule PC nuclear DAPI staining was compared with that of DAPI-stained haploid (1C) spermatid nuclei from dissected adult testes as previously described (Losick et al., 2013). Briefly, testes and renal tubules were fixed, stained and imaged in tandem with identical confocal settings and images were analyzed using integrated fluorescence intensity measurements in ImageJ. For exposure to exogenous stress, adult *Drosophila* previously raised in vials on standard food were transferred to an empty vial without food for 16 h (for desiccation stress conditions) or raised on standard food supplemented with 1% H_2_O_2_ for 16 h (for oxidative stress conditions) before confocal imaging. For imaging of adult intact *Drosophila*, adults were briefly anaesthetized with carbon dioxide before imaging using a brightfield-equipped dissecting scope (Motic).

For analysis of mitochondrial density, maximum *z*-projections were generated (ImageJ), followed by thresholding to measure the mitochondrial area and division by the total cell area. For analysis of JC-1 and ATP Red in PCs and SCs, the average fluorescence intensity of a SC and a neighboring PC were obtained from maximum *z*-projections. A similar method was used for quantification of relative mitochondrial redox state (using *mito-roGFP2-Grx1*) with ratiometric images generated as described above. For quantification of mitochondrial activity (JC-1 staining) between different developmental stages, the intensity of 10×56 pixel^2^ regions along the MpT were measured using maximum *z*-projections. Similarly, for quantification of relative mitochondrial redox state, maximum *z*-projection ratiometric images were generated (as described above), and an average 405:488 intensity ratio was calculated (10× sampled regions not required as ratiometric images have ‘Not-a-number’ values assigned to background pixels). For both JC-1 and ratiometric analysis, three images of different sections of the MpT per fly were imaged.

### Immunostaining and *in situ* hybridization

Immunostaining was performed using standard techniques (Rebay and Fehon, 2008) with the following antibodies and reagents: anti-γH2AvD (rabbit, GeneTex, GTX48733, 1:500), anti-8-oxodG (mouse, Trevigen, clone 15A3, 1:200), anti-Cut (mouse, 2B10, Developmental Studies Hybridoma Bank, 1:200), anti-CC3 (rabbit, Cell Signaling Technology, 5A1E #9664, 1:50), anti-Fascin (mouse, sn1c, Developmental Studies Hybridoma Bank, 1:50), anti-Futsch (mouse, 22C10, Developmental Studies Hybridoma Bank, 1:200), anti-GFP (goat, Abcam, ab6673, 1:500), anti-RFP (rabbit, MBL, PM005, 1:500), Phalloidin-Alexa-Fluor-633 (Invitrogen, 1:100) and DAPI (Invitrogen, D1306, 1:100). An extra amplification step was performed where required using biotinylated secondary antibodies (Vector Laboratories, horse anti-mouse, 1:200; BA-2000-1.5) and Streptavidin-conjugated fluorophores [Fluorescein (DTAF) Streptavidin, 016-010-084, Jackson ImmunoResearch]. Carefully staged embryos were oriented and mounted on a glass slide in Vectashield (Vector Laboratories) and imaging was performed on a Leica SP5 confocal microscope. *Gadd45* RNA localization was performed by *in situ* hybridization using DIG-labeled RNA probes generated by *in vitro* transcription from cDNA templates (RE38191, Berkeley *Drosophila* Genome Project). Hybridization and staining was performed according to standard protocols (Tautz and Pfeifle, 1989).

### Analysis of adult *Drosophila* physiology

To quantify the extent of adult abdominal bloating and measure wet and dry body weights, groups of ten 1-week old adult (male or female) flies were briefly anaesthetized with CO_2_ and ‘wet’ weights measured in ice-cooled Eppendorf tubes using a precision balance. Flies were sacrificed by freezing (30 min) and incubated for 48 h in a drying chamber with silica crystals (for desiccation) before repeating the measurements to obtain the ‘dry’ weights for each experimental group as previously described (Denholm et al., 2013).

Fluid secretion assays (also known as Ramsay Assays) were performed as previously described (Dow et al., 1994). Briefly, live MpTs were dissected from control *OrR* flies (stage dependent on experiment) in ice-cold Schneider's medium (Sigma-Aldrich). MpTs were transferred to Schneider's filled wells in custom-made assay plates (Schellinger and Rodan, 2015), topped with a layer of paraffin oil (Sigma-Aldrich). One MpT was wrapped around an insect pin, while the other MpT remained in the well; fluid droplets accumulating at the ureter (in paraffin oil) were collected at 60 min intervals, from which droplet volume, and thus secretion rate, could be calculated.

## Supplementary Material

Supplementary information

Reviewer comments
