# Supplementary material for: Metabolically active and polyploid renal tissues rely on graded cytoprotection to drive developmental and homeostatic stress resilience
Source: Development. 2021 Apr 26;148(8):dev197343. doi: 10.1242/dev.197343 (PMC8214761; doi:10.1242/dev.197343)
Supplement: Supplementary information [file develop-148-197343-s1.pdf]

Figure S1

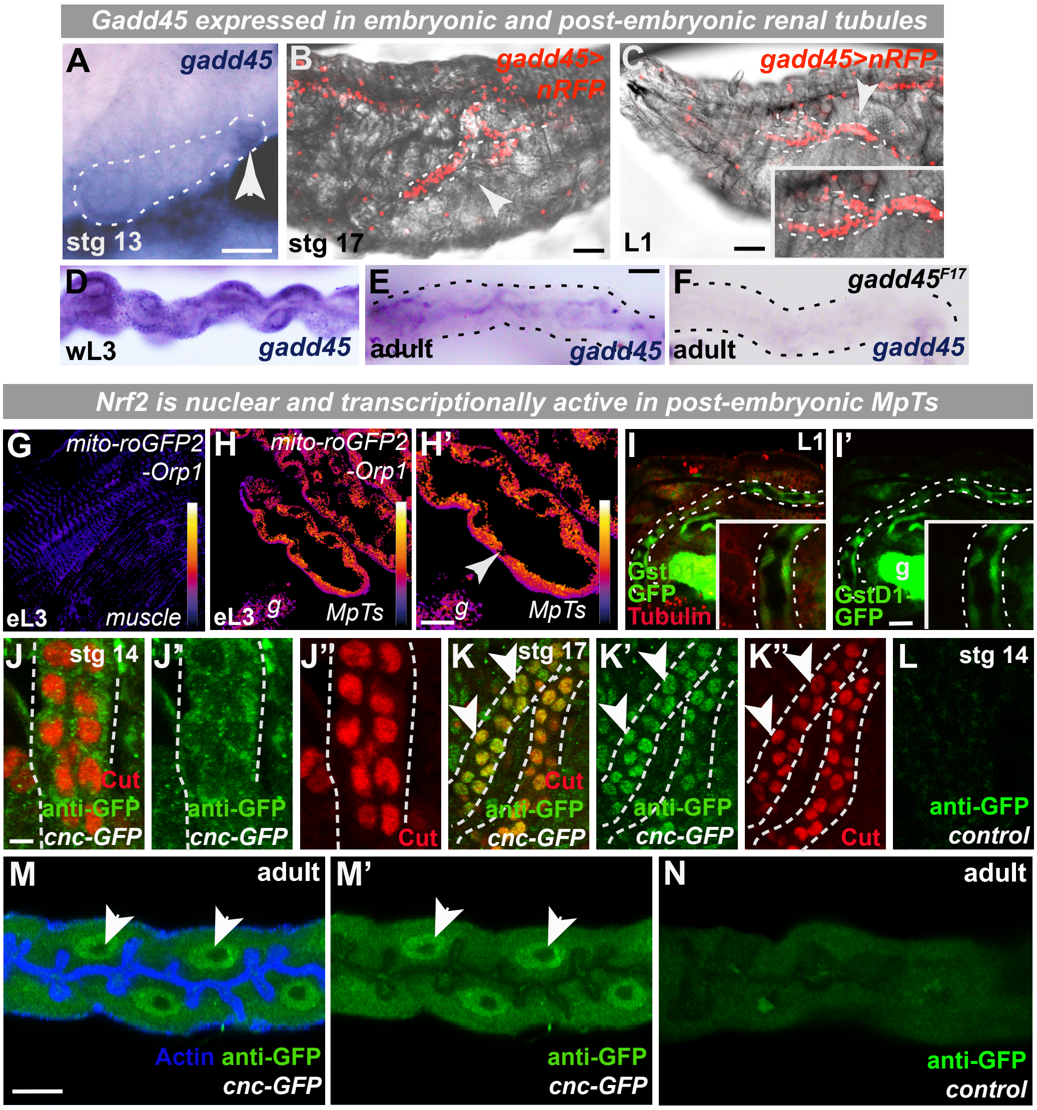

Figure S1. Cytoprotective genes Gadd45 and Nrf2 are active *in vivo* during renal tubule development and homeostasis

Gadd45 expression during embryonic MpT development (A, arrow indicates distal tip cell; *in situ* hybridisation), in both MpT pairs by embryonic stage 17 (arrow, B; red, *gadd45>nRFP*) and into post-embryonic life (arrow, C-E; *in situ* hybridisation). *gadd45* expression absent from amorphic *gadd45*^{F17} mutant MpTs (F). Physiologically active MpTs are more oxidised than surrounding tissues (e.g. muscles) as shown by the ratiometric ROS sensor *mito-roGFP2-Orp1* (G-H). Nrf2 (green, GstD1-GFP) is active within MpTs from the onset of physiological activity (1st larval instar, I). Nrf2 (green, GFP-tagged *Drosophila cnc*) localisation in embryonic MpTs (J-K, arrows indicate nuclear GFP; L, no GFP negative control) and adult MpTs (M, arrows indicate nuclear GFP; N, no GFP negative control). Scale bars represent 40μm (panels A-C, I-K, M-O and I) and 20μm (D-E, G-H, J and M). N numbers: >10 animals or >10 tubules from independent hosts examined per developmental stage per experiment.
See also Figure 1 and Movie S1.

Figure S2

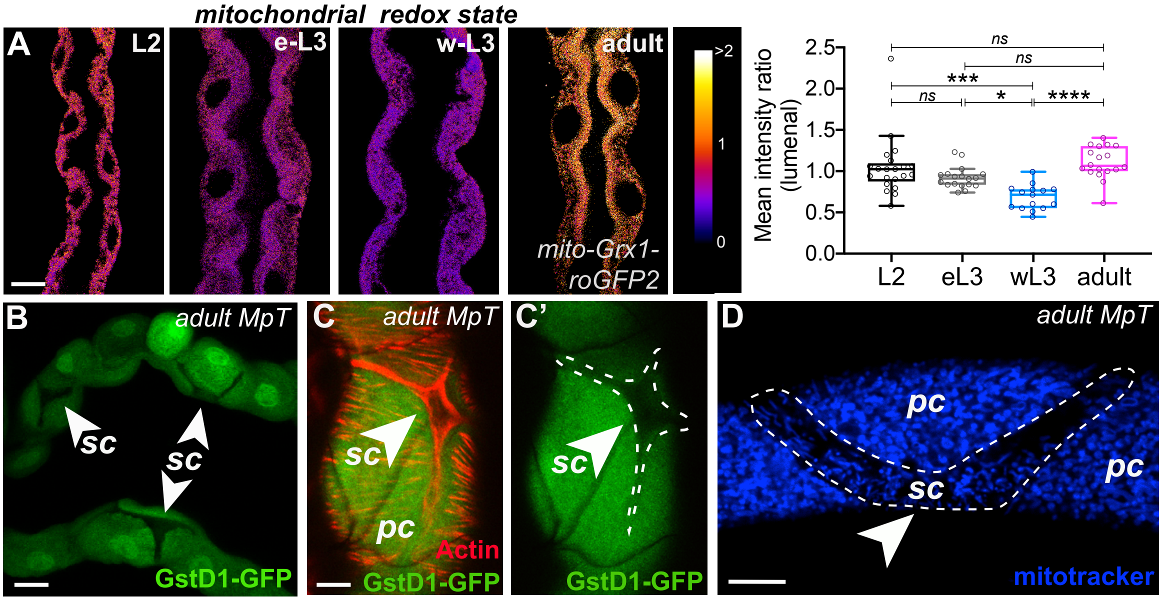

Figure S2. Nrf2 activity enriched in metabolically active renal PCs

Relative mitochondrial redox state in MpTs during development correlates with a drop off in secretory activity towards the pupal stage that returns before adulthood (A, ratiometric *mito-roGFP2-Grx1*; quantification performed on z-sections cutting through the MpT lumen). Nrf2 activity (green, GstD1-GFP and red, Actin) in principal and stellate cells (sc, indicated by arrowheads or dashed line) of adult MpTs. Mitochondria in stellate (sc, dashed line) and principal (pc) cells (D; blue, MitoTracker) of adult MpTs. Scale bars: 20μm (A, B and D) and 10μm (C). Data represented as box and whisker tukey plots with individual MpT sections shown as overlaid points; *ns, not significant*, *$p < 0.05$, ***$p < 0.001$, ****$p < 0.0001$ via one-way ANOVA with multiple comparisons. N numbers: 7 L2, 6 eL3, 5 wL3 and 6 1-week adults (A), >10 tubules (B-C) and tubules from 5-8 flies, 12-33 cells (D).
See also Figure 2.

Figure S3

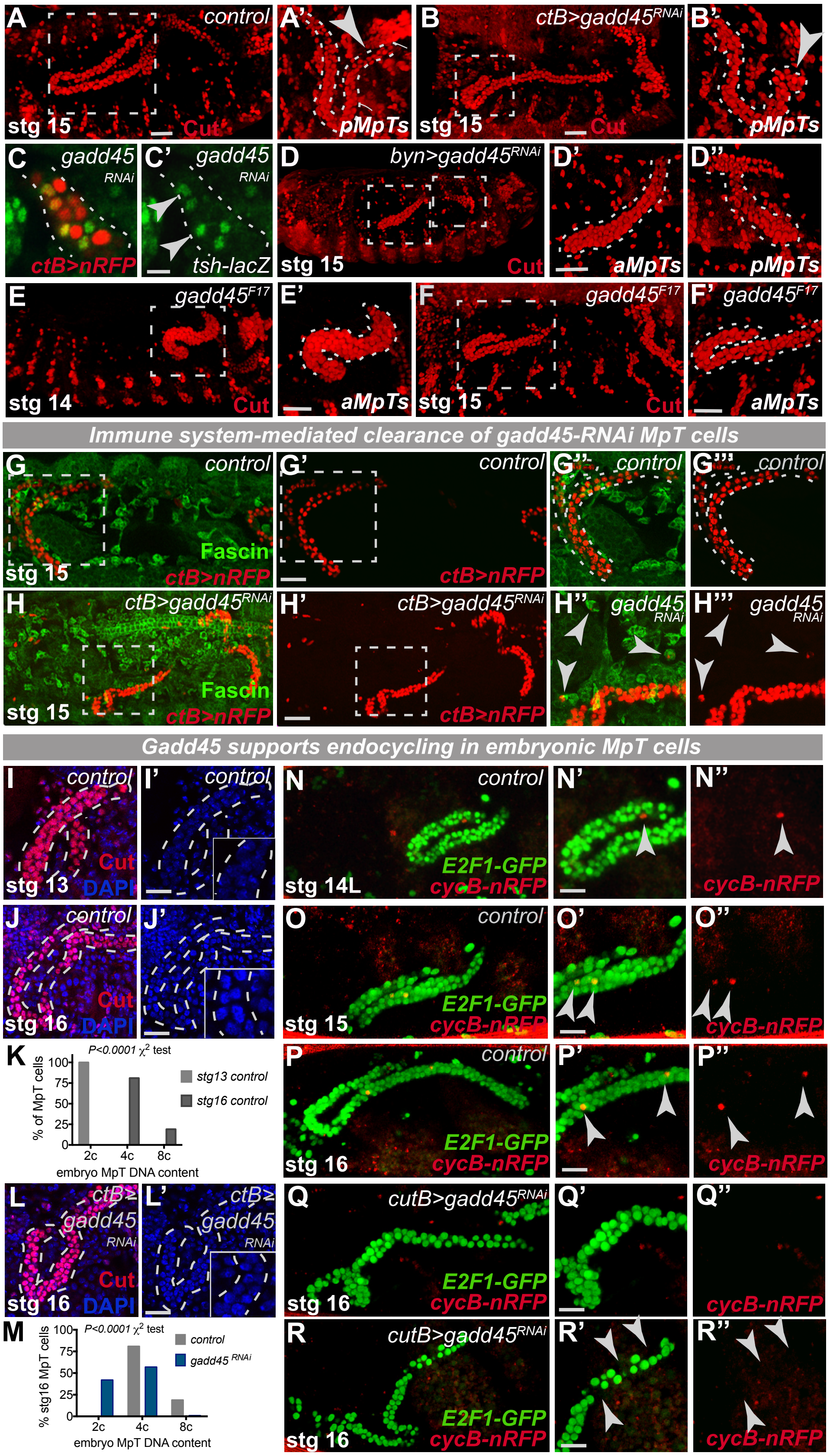

Figure S3. Gadd45 is essential for embryonic MpT morphogenesis *in vivo*

MpT morphogenesis (red, Cut) is abnormal following tubule-localised expression of *gadd45-RNAi* (using multiple independent Gal4 drivers, A-D) but stellate cells (arrows, C; *tsh-lacZ*, green) are present as in *control* MpTs. Similar MpT defects are observed in *Gadd45*^{F17} mutant embryos (E-F). Hemocytes (green, Fascin) from *control* embryos do not contain Cut-positive corpses (G) but Cut-positive corpses (red, *ctB-gal4* driven *nRFP*) are abundant in hemocytes following tubule-specific *gadd45-RNAi* (arrowheads, H). Control MpT cells endocycle from stage 13 onwards, increasing DNA content (I-K) and moving through S-phase (red, *cyclinB-nRFP*; arrowheads, N-P). MpTs lacking Gadd45 have lower ploidy by stage 16 (L-M) and reduced *cyclinB-nRFP* (Q-R). Gaps in Fucci staining are apparent in MpTs lacking Gadd45 (arrowheads, R). Scale bars represent 20μm (A-B, D-J, L and N-R) and 10μm (C). For statistical analysis, *p<0.0001* via χ^2 tests (K and M). N numbers: >15 embryos (A-F) and >10 embryos (G-R) examined per genotype, condition or developmental stage. See also Figure 3 and Movies S2-S3.

Figure S4

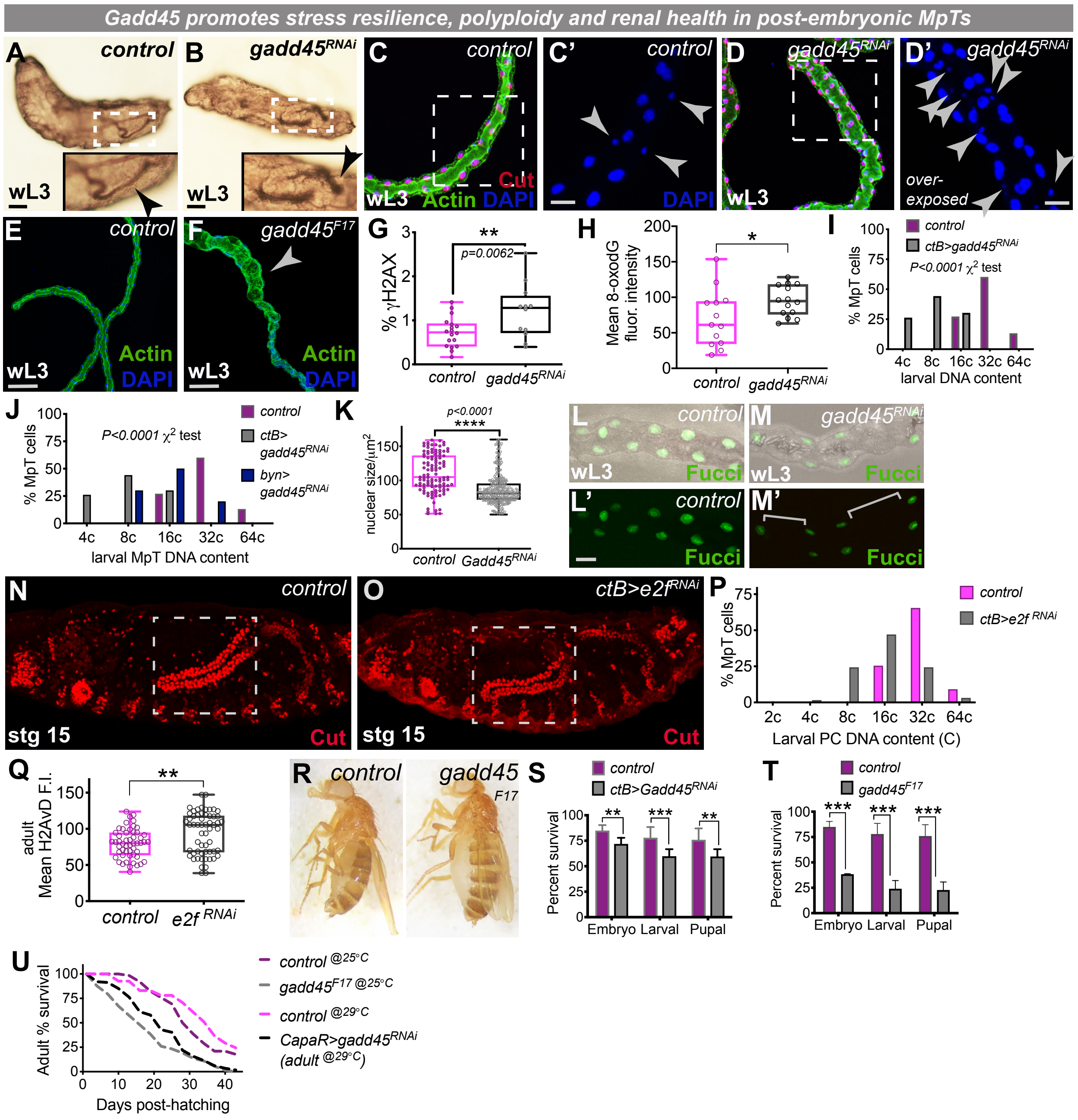

Figure S4. Gadd45 drives stress resilience during MpT homeostasis

Unlike the long MpTs of control larvae (arrowhead, A) with only 2 PCs around the MpT lumen (C) and stellate cells evenly spread (arrowheads, C'), MpTs lacking Gadd45 are shorter than controls (B) with multiple PCs around the lumen (D) and stellate cells located in uneven clusters (arrowheads, D'). Similar MpT phenotypes are observed in *gadd45^{F17}* mutants (E-F) with abnormal enlargements (arrowhead, F). *ctB>gadd45-RNAi* L3 MpTs exhibit higher levels of DNA damage (γH2AvD staining, G) and oxidative damage (H), reduced ploidy (DAPI staining, I-J), significantly smaller nuclei (K) and conspicuous lack of *E2F1-GFP* (from Fucci) along the MpT length (L-M). *RNAi*-mediated inhibition of tubule *e2f* did not perturb embryonic MpT development (N-O) but reduced larval PC ploidy (P) and increased adult DNA damage signalling (Q). Adult hosts lacking tubule Gadd45 suffer abdominal bloating (R) and reduced survival (S-U, n>50 per experimental group; log-rank test statistic *$p<0.001$* for control$^{25°C}$ vs *gadd45^{F17}* and control$^{29°C}$ vs *capaR>gadd45-RNAi* $^{\text{adult only}}$). Data represented as box and whisker plots with all data shown as overlaid points, bar graphs or line graphs; *ns, not significant, *p < 0.05, **p < 0.01, ***p < 0.001, ****p < 0.0001*, via unpaired t-tests (followed by Holm-Sidak multiple comparisons correction where appropriate), χ^2 tests or Log-rank survival analyses (U). Scale bars represent 20μm (C-D and L-M) and 80μm (E-F). N numbers: >10 larvae or tubules (A-F and L-M), >12 tubules (G), >10 tubules (H), >30 PCs from >6 tubules (I-K and P-Q), >15 embryos (N-O) and >100 animals (S-U) examined per genotype, condition or developmental stage.

See also Figure 4.

Development • Supplementary information

Figure S5

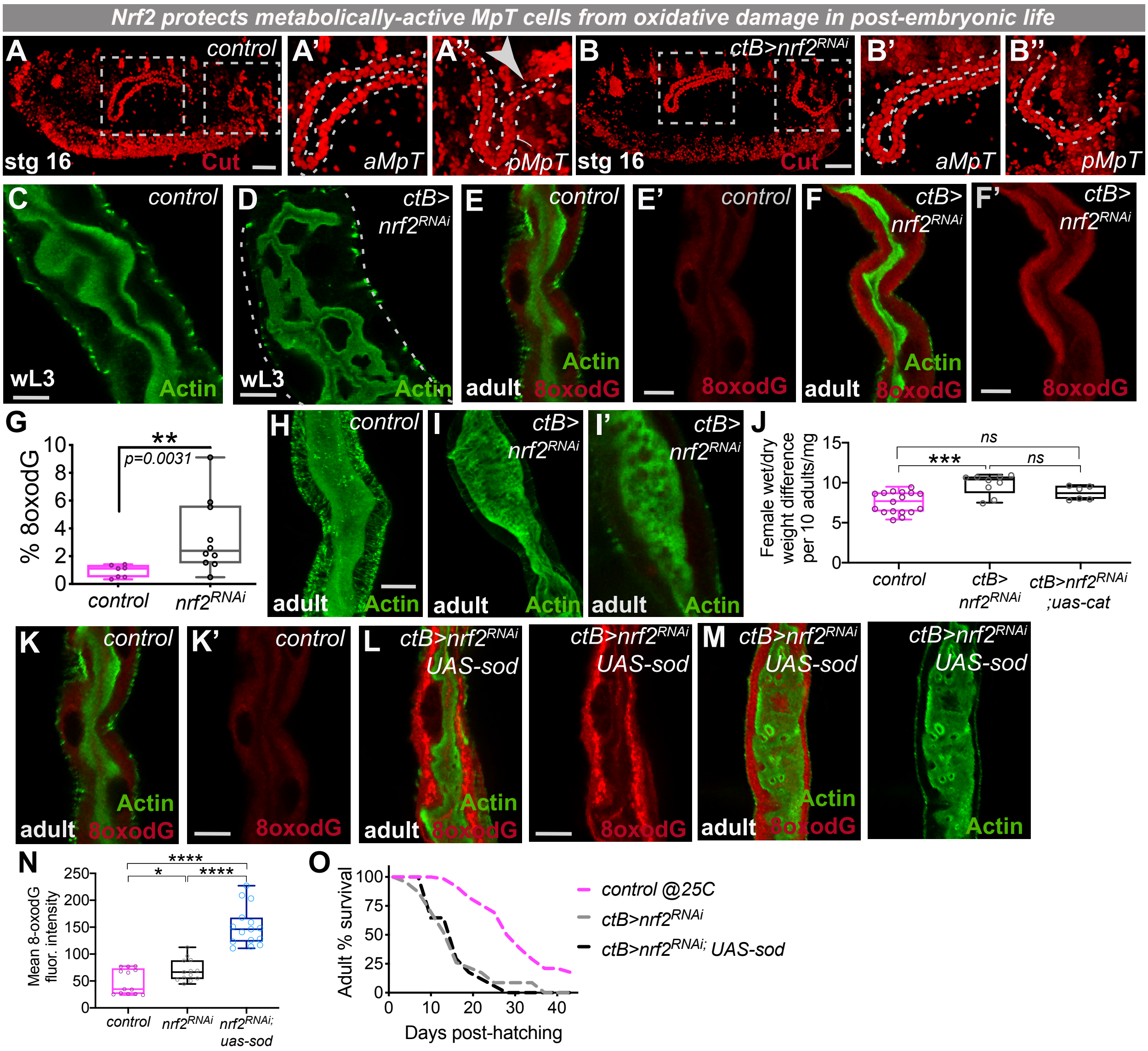

Figure S5. Nrf2 drives oxidative stress resilience during MpT homeostasis
Nrf2 is not required for embryonic MpT development (red, Cut staining; A-B) but larval MpTs lacking Nrf2 possess abnormally convoluted and dilated lumens (C-D), increased oxidative DNA damage (E-F, adults and G, L3 MpTs) and luminal defects persist into adulthood (H-I). Expression of catalase reduces the mean wet/dry weight measurements of *ctB>nrf2-RNAi* adults (although this is not statistically significant) but expression of *sod2* exacerbates the oxidative damage (K-N) and does not rescue adult survival (O; log-rank statistic of *p=0.17* for *ctB>nrf2-RNAi* vs *ctB>nrf2-RNAi;UAS-sod2*). Scale bars represent 100μm (panels A-B) and 20μm (panels C-F, H-I and K-M). Data represented as box and whisker plots with all data shown as overlaid points or line graphs; *ns, not significant, *p < 0.05, **p < 0.01, ***p < 0.001, ****p < 0.0001*, via unpaired t-tests (followed by Holm-Sidak multiple comparisons correction where appropriate) or Log-rank survival analyses (O). N numbers: >10 embryos (A-B), >10 tubules (C-I and K-N), >100 adults (J and O) examined per genotype, condition or developmental stage. See also Figure 5.

Development • Supplementary information

Figure S6

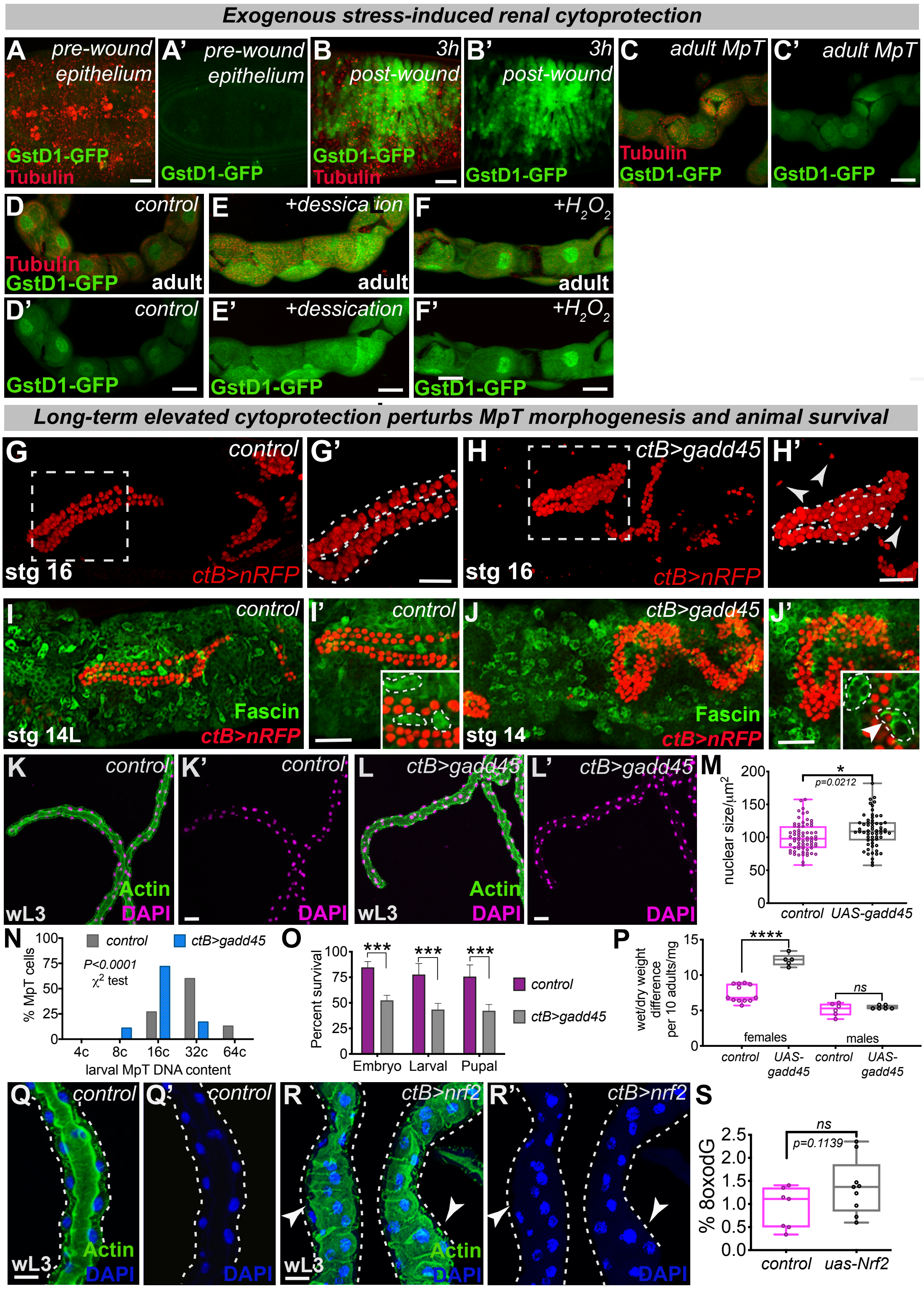

Figure S6. Elevated cytoprotection confers increased stress resilience but is detrimental to MpT morphogenesis and host survival

Nrf2 activity in adult MpTs (green, GstD1-GFP) *versus* unwounded epithelial tissue and repairing epithelial tissue following wounding (A-C; red, Tubulin). Adult MpT Nrf2 activity in response to exogenous stress (D-F, dessication or H_2O_2 treatment; green, GstD1-GFP and red, Tubulin). Ectopic *gadd45* expression in embryonic MpTs (*ctB>gadd45*) disrupts MpT morphogenesis (G-H) and Cut positive corpses (red, *ctB>nRFP*) are observed within hemocytes (green, Fascin; arrowhead, J compared to control, I). Post-embryonic MpTs with elevated Gadd45 are misshapen, with more PCs around the MpT lumen (K-L), PC nuclei are larger (M) with reduced ploidy (N), but hosts exhibit reduced host survival (O) and bloating (P). Experimental elevation of tubule Nrf2 also leads to abnormal MpT morphology (Q-R), with enlarged regions along the MpT length (arrowheads, R) and a small but non-significant rise in oxidative DNA damage (S). Scale bars represent 10μm (A-B), 20μm (C-F and Q-R) and 40μm (G-L). Data represented as box and whisker plots with all data shown as overlaid points or bar graphs; *ns, not significant, *p < 0.05, **p < 0.01, ***p < 0.001, ****p < 0.0001*, via unpaired t-tests or χ^2 tests. N numbers: >10 tubules or embryos (A-J), >30 PCs from >6 tubules (K-N), >100 animals (O-P) and >7 tubules (Q-S) examined per genotype or condition. See also Figure 6.

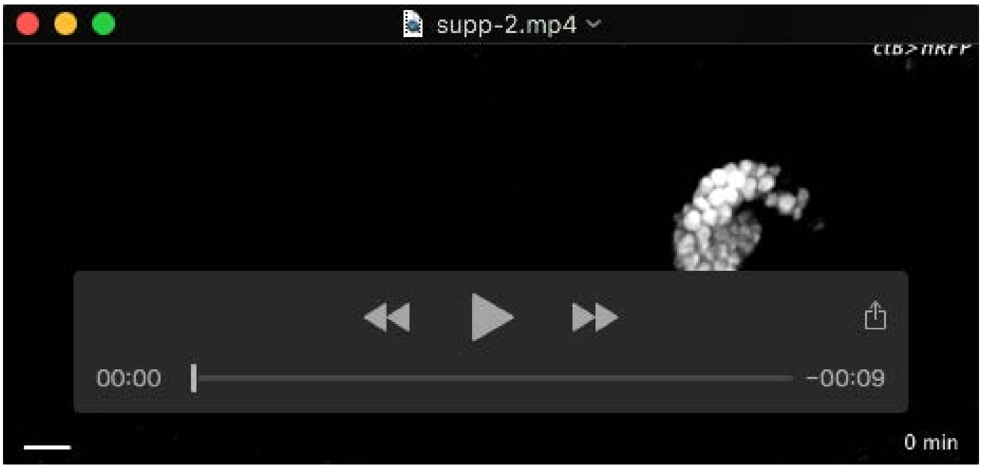

Movie 1: Live-imaging of wild-type embryonic renal tubule morphogenesis

Time-lapse *in vivo* imaging of embryonic (anterior) renal tubule morphogenesis from stage 13 onwards using *ctB-gal4* driven expression of nRFP. By stage 13 of embryogenesis, the renal tubules have reached their mature cell number and commence their stereotypical rearrangements (involving convergent extension movements) and navigation to reach highly reproducible 3-D arrangements in the body cavity by stage 16. Z-stacks were taken at regular 4 minute time intervals. Scale bar represents 20µm. See also Figure 1 and Figure S1.

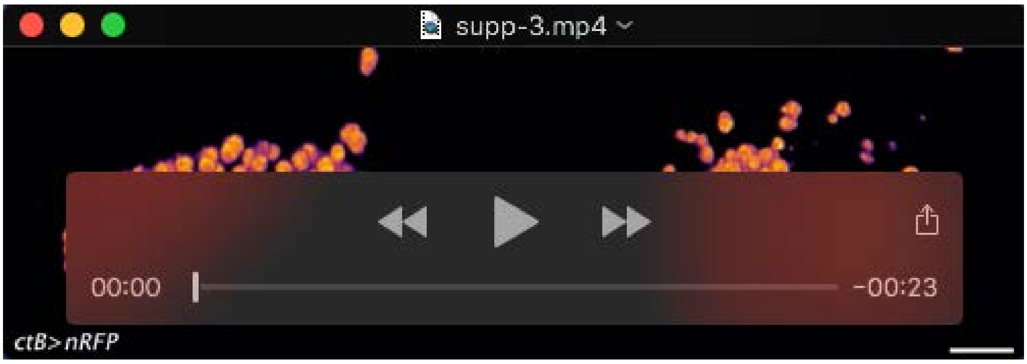

Movie 2: 3-D rotations of *control* and *gadd45-RNAi* embryonic renal tubules

Live *in vivo* imaging of control (left) and *ctB>gadd45-RNAi* (right) anterior renal tubules at embryonic stage 14, where tubule cell nuclei are labelled using *ctB-gal4* driven expression of nRFP. 3-D rotations around the renal tubules *in vivo* illustrates the differences in morphology between control tubules and those lacking Gadd45; whilst no Cut positive tubule nuceli are visible outside of the control tubules (left panel), significant numbers of Cut positive nuclei are observed outside of the misshapen tubule lacking Gadd45 expression. Scale bar represents 20µm. See also Figure 2 and Figure S2.

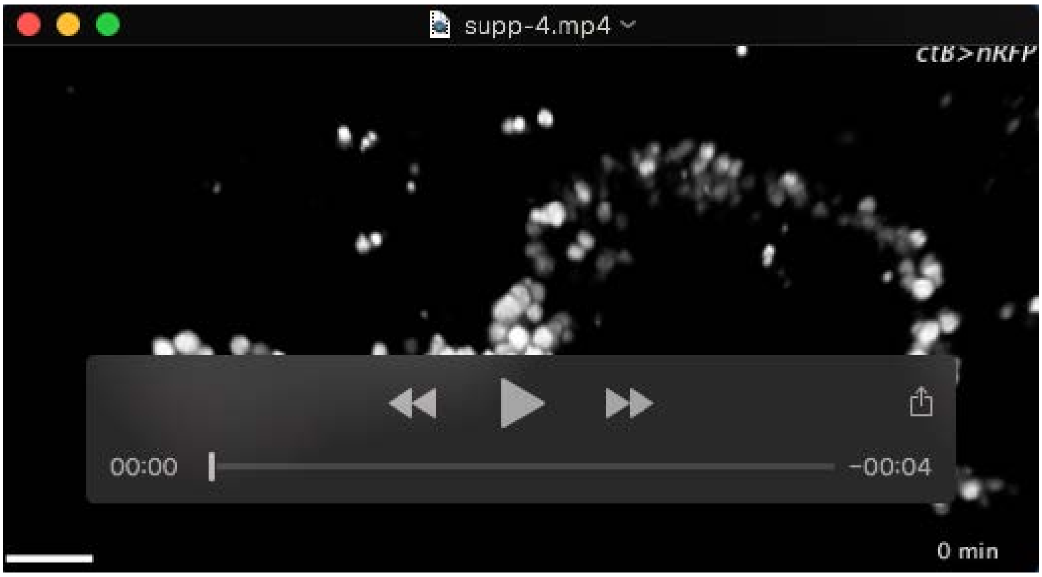

Movie 3: Live-imaging of *gadd45-RNAi* renal tubule morphogenesis

Live *in vivo* imaging of *ctB>gadd45-RNAi* anterior renal tubules at embryonic stage 14, where tubule cell nuclei are labelled using *ctB-gal4* driven expression of nRFP. A RFP-positive tubule nucleus can be observed leaving the anterior tubule (arrowhead); many other RFP-positive punctae are observed moving within the extracellular space around the developing tubules which we envision to be tubule cell corpses contained within migratory hemocytes. Scale bar represents 20µm. See also Figure 2 and Figure S2.

Development • Supplementary information
