# Supplementary material for: Metabolically active and polyploid renal tissues rely on graded cytoprotection to drive developmental and homeostatic stress resilience
Source: Development. 2021 Apr 26;148(8):dev197343. doi: 10.1242/dev.197343 (PMC8214761; doi:10.1242/dev.197343)
Supplement: Reviewer comments [file develop-148-197343_review_history.pdf]

Metabolically active and polyploid renal tissues rely on graded cytoprotection to drive developmental and homeostatic stress resilience

Katie Burbridge, Jack Holcombe and Helen Weavers
DOI: 10.1242/dev.197343

Editor: Irene Miguel-Aliaga

Review timeline
Original submission: 14 October 2020
Editorial decision: 9 November 2020
First revision received: 1 March 2021
Editorial decision: 22 March 2021
Second revision received: 25 March 2021
Accepted: 29 March 2021

Original submission

First decision letter

MS ID#: DEVELOP/2020/197343

MS TITLE: Graded cytoprotective activity drives long-term stress resilience within vulnerable renal cell types during development and homeostasis

AUTHORS: Katie Burbridge and Helen Weavers

I have now received all the referees' reports on the above manuscript, and have reached a decision. The referees' comments are appended below, or you can access them online: please go to BenchPress and click on the 'Manuscripts with Decisions' queue in the Author Area.

As you will see, the referees express considerable interest in your work, but have some significant criticisms and recommend a substantial revision of your manuscript before we can consider publication.If you are able to revise the manuscript along the lines suggested, I will be happy to receive a revised version of the manuscript. Your revised paper will be re-reviewed by one or more of the original referees, and acceptance of your manuscript will depend on your addressing satisfactorily the reviewers' major concerns. Please also note that Development will normally permit only one round of major revision.

In particular, the three following points should be addressed in a revised version of the manuscript:

Provide additional data that strengthens the link between active transport and oxidative stress
Uncouple the developmental and physiological effects of Nrf2 and Gadd45 with temporally confined downregulations
Investigate a possible epistatic relationship between Nrf2 and Gadd45

We are aware that you may be experiencing disruption to the normal running of your lab that make experimental revisions challenging. If it would be helpful, we encourage you to contact us to discuss your revision in greater detail. Please send us a point-by-point response indicating where you are able to address concerns raised (either experimentally or by changes to the text) and

© 2021. Published by The Company of Biologists under the terms of the Creative Commons Attribution License (https://creativecommons.org/licenses/by/4.0/).

where you will not be able to do so within the normal timeframe of a revision. We will then provide further guidance. Please also note that we are happy to extend revision timeframes as necessary.

Please attend to all of the reviewers' comments and ensure that you clearly highlight all changes made in the revised manuscript. Please avoid using 'Tracked changes' in Word files as these are lost in PDF conversion. I should be grateful if you would also provide a point-by-point response detailing how you have dealt with the points raised by the reviewers in the 'Response to Reviewers' box. If you do not agree with any of their criticisms or suggestions please explain clearly why this is so.

Reviewer 1

Advance summary and potential significance to field

The kidney is continuously exposed to various stressors, and oxidative stress plays a key role in the development of chronic kidney disease. Therefore, this tissue requires particular efficient stress defense strategies. Based on previous data of the laboratory in skin wounding models, the authors hypothesized that the cytoprotective Nrf2 transcription factor as well as the stress response protein Gadd45, which is involved in DNA repair and cell cycle control, may also play critical roles in the kidney - even under physiological conditions. Indeed, this study shows that a tight regulation of both proteins is required to prevent kidney malformation and damage. Although it has previously been shown that a tight regulation of these proteins is required for normal tissue development and homeostasis, their roles in the developing kidney have not been characterized in detail. The results are convincing and important for the fields of kidney development and clinical nephrology and they also contribute to the general understanding of the function of both stress proteins.

Comments for the author

1.) The paper convincingly shows important functions of Gadd45 and Nrf2 in kidney development and homeostasis. Although both are stress-regulated proteins, the link between Nrf2 and Gadd45 is not clear.
As it stands, the paper reports two different stories. Therefore, it would be important to know if Gadd45 gain- or loss-of-function affects Nrf2 expression/activity and vice versa. Do they have some overlapping functions?

2.) The authors should add a paragraph to the introduction in which they introduce Gadd45 and Nrf2 and their regulation. This is important information for the non-specialist.

3.) The Nrf2 reporters allow the analysis of Nrf2 activity, but their sensitivity is not particularly high. It would be interesting to know if Nrf2 is indeed not expressed in embryonic MpTs or if it is simply not efficiently activated.

4.) Fig, 2H-L: The number of gH2Avd and cleaved caspase-3 positive cells and the number of hemocytes with corpses should be quantified.

5.) Fig. 3A-F: The abnormalities in larval and adult life may well be a consequence of the embryonic defects and thus may not reflect the role of Gadd45 at these later stages. This should be at least discussed.

6.) Page 9, end of last paragraph: Here, I would also cite Tsakiri et al., 2019, who showed negative consequences of Nrf2 overexpression in Drosophila. In particular, the authors should cite the paper by Suzuki et al., 2017 in this context (showing adverse effect of constitutive Nrf2 activation in the mouse kidney).

7.) Please mention the number of flies analyzed for each figure and supplementary figure in the legends even if no statistical analysis is presented (e.g. representative of ...).

8.) Some references that are in the reference list are not cited in the text and some references that are cited in the text are not in the reference list - please double check.

© 2021. Published by The Company of Biologists under the terms of the Creative Commons Attribution License (https://creativecommons.org/licenses/by/4.0/).

Reviewer 2

Advance summary and potential significance to field

In this manuscript, Burbridge and Weavers examine the role of two cytoprotective genes, gadd45 and Nrf2, in Malpighian tubule development, function and stress responses. Gadd45 is expressed early beginning in embryogenesis, whereas Nrf2 activity is largely absent during embryogenesis and then begins during the larval period, at the onset of transport function. MpTs have higher levels of oxidative stress than other tissues, particularly in the metabolically active principal cells. Loss of gadd45, which is assessed by MpT PC RNAi as well as a loss-of-function mutant, results in developmental abnormalities in the tubules, DNA damage, apoptosis, and delamination of principal cells with hemocyte phagocytosis. In addition, there is decreased endocycling and ploidy of PC nuclei. Stones appear in the tubule lumen edema develops, and there is decreased survival. Loss of Nrf2 in PCs also causes morphological abnormalities, oxidative stress, edema and decreased survival. Gadd45 and Nrf2 overexpression also results in morphological abnormalities, decreased ploidy, edema, and decreased survival, indicating that both decreased and increased expression of these cytoprotective genes are detrimental. These findings advance understanding of stress response mechanisms in the normal development and physiological function of Malpighian tubules, an interesting tissue given high metabolic demands to support transport function, as well as their role in detoxification and waste excretion.

Comments for the author

Suggested revisions.

1. The authors propose that actively transporting tubules are more metabolically active and therefore have higher oxidative stress – interesting idea, and makes sense. This case could be further bolstered by examining tubules during periods of decreased transport: wandering L3/pupae. (Hard to find published data on this, but anecdotally transport shuts down during these periods. Might be nice to get someone to do Ramsay assays on tubules from these stages to get the data published and correlate with oxidative stress.)
2. Principal cell polyploidy is intriguing, as is decreased ploidy with loss of gadd45. Given the statement that “polyploidy may be a conserved mechanism for promoting stress resilience in vulnerable tissues,” is it possible to decrease ploidy through some other means, and examine effects on tubules? This would strengthen the argument for a role for ploidy in physiological stress resistance.
3. The manuscript shows related phenotypes for decreased and increased gadd45 and Nrf2. However the relationship between gadd45 and Nrf2 is not examined. For example, does loss of gadd45 result in Nrf2 activation?

Minor comments.

1. Figure 1 B-E. Please state in figure legend what dotted white line is encircling.
2. Figure 1 H,H’. Please label stage. Looks like L3 but doesn’t say. Also, please indicate what part of L3 experiments were done in, given comment above about transport in early vs. wandering L3.
3. Figure 1 N-P. Please indicate in figure legend what arrowheads are pointing to. Looks like stellate cells but doesn’t say.
4. Figure 2 M-N. Would be nice to (optionally) show quantification of the Fucci experiments.
5. Figure 3 N, W and Figure S3 S. Are the control and gadd45/Nrf2 RNAi curves statistically different?
The text says “tubule defects were associated with… significantly shorter lifespans than controls.” Similarly for Figure 4K, S.
6. Figure S3. In adults, byn-GAL4 is expressed in hindgut and not in Malpighian tubules. How late in development is byn-GAL4 expressed in the tubules?

© 2021. Published by The Company of Biologists under the terms of the Creative Commons Attribution License (https://creativecommons.org/licenses/by/4.0/).

Reviewer 3

Advance summary and potential significance to field

In this paper, the authors described a constitutive protective role of Gadd45 and Nrf2 in Drosophila Malpighian tubules principal cells. They found that physiologically-active Malpighian tubules were more oxidized with or without exogenous challenge. Knocking down Gadd45 led to DNA damage, apoptosis, affected immune clearance, and endocycling. Both Gadd45 and Nrf2 knocking down shortens the lifespan. Lastly, they found that these protective activities need to be tightly controlled to a modest level. While the topic itself is interesting, several major problems weakens the conclusion of the paper and needs to be addressed.

Comments for the author

1. Background knowledge on Gadd45 and Nrf2 should be included in the introduction part.
2. It is confusing that the authors mixed results and discussion part. To improve the readability of the paper, it would be better to separate these two parts.
3. The authors acclaimed that Gadd45 and Nrf2 are required both in development and also during homeostasis. However, they failed to detangle developmental and the adult flies' phenotype. Without this, it is difficult to know the real reason for the adult phenotype. For example, are the shortened lifespan and bloating abdomen in Gadd45 and Nrf2 knocking down fly due to the malformation of tubules during development or the malfunction of adult tubules? They should use Gal80TS lines to analyse the physiological of silencing Gadd45 and Nrf2 only in adults.
4. ctb-Gal4 is a good driver for embryogenic research; it is not the best driver to study adult phenotype. Using other principal cell drivers (uro-Gal4, or capaR-Gal4) combined with Gal80 system would answer both point 3 and point 4.
5. It is difficult to link constitutive renal cryoprotection with Gadd45 based on current data. Current data only suggested Gadd45 is constitutively expressed throughout the fly's life cycle. However, the rest data in Figure 1 and S1 only provided the evidence linking nrf2 with excessive ROS and stress challenge in the tubules. Does Gadd45 also respond to exogenous stress challenges like Nrf2? Does Gadd45 also protect tubules from oxidative stress like Nrf2?
6. It seems like Nrf2 is required to control the excess ROS in the tubules. Can authors rescue the phenotype by reducing ROS? It would be great to know how Gadd45 and Nrf2 mediate their protective role.

First revision

Author response to reviewers' comments

"Metabolically active and polyploid renal tissues rely on graded cytoprotection to drive developmental and homeostatic stress resilience"

In summary, as requested by multiple reviewers, we have performed new experiments to uncouple the developmental and physiological roles of Nrf2 and Gadd45 with temporally confined downregulations during post-embryonic and adult life, as well as exploring whether Gadd45 and Nrf2 have overlapping roles (or impact on each other's expression or activity). We have also extended our study by providing additional data that strengthens the link between renal physiological (secretory) activity and oxidative stress. We have tested whether polyploidy has roles in renal stress resilience, performed additional quantification of the embryonic *gadd45-RNAi* phenotypes and tested whether expression of ROS scavengers can rescue the renal *nrf2-RNAi* phenotypes. Finally, as suggested by Reviewer 3 and the Editorial Administrator (communication with Laetitia Beck on 27/11/20), we have reformatted our revised manuscript as an Article, which was very helpful because we felt that our original manuscript was at the 'crowded' end of a Report and with these additional revision data it would have been over full.

We are grateful for the reviewers' very helpful suggestions that led us to perform these additional experiments and quantification, which we feel have significantly improved the paper. The text

© 2021. Published by The Company of Biologists under the terms of the Creative Commons Attribution License (https://creativecommons.org/licenses/by/4.0/).

below provides a detailed response to individual reviewer comments with our response to each suggestion/comment highlighted in bold following the text of their reviews. We have also highlighted each individual revision within the revised manuscript in yellow.

Reviewer #1:
1) The paper convincingly shows important functions of Gadd45 and Nrf2 in kidney development and homeostasis. Although both are stress-regulated proteins, the link between Nrf2 and Gadd45 is not clear. As it stands, the paper reports two different stories. Therefore, it would be important to know if Gadd45 gain- or loss-of-function affects Nrf2 expression/activity and vice versa. Do they have some overlapping functions?
We have now explored the relationship between Nrf2 and Gadd45 within the renal tubules and analysed whether they perform overlapping functions (Figures 4-6). Intriguingly, both Nrf2 and Gadd45 are required to protect renal cells from oxidative (8-oxodG) and DNA damage (□H2AX) (Figure 5C-I, Figure 4G-H and Figure S4G-H). We envision that the increased oxidative damage observed following *gadd45-RNAi* could reflect the increased inflammatory response in these animals (Figure 3); indeed, we find that loss of *gadd45* also elevated Nrf2 activity within the renal tubules (Figure 6E). Our data also suggest that Nrf2 can feed back on and promote Gadd45 expression, since ectopic Nrf2 expression led to increased renal Gadd45 levels (Figure 6D). Finally, we find that both Nrf2 activity and Gadd45 expression are elevated by exogenous stress (Figure 6B-C).

2) The authors should add a paragraph to the introduction in which they introduce Gadd45 and Nrf2 and their regulation. This is important information for the non-specialist.
We agree and have now included more detail about Nrf2 and Gadd45 within the Introduction (pages 3-4).

3) The Nrf2 reporters allow the analysis of Nrf2 activity, but their sensitivity is not particularly high. It would be interesting to know if Nrf2 is indeed not expressed in embryonic MpTs or if it is simply not efficiently activated.
We have now analysed the levels of Nrf2 protein using GFP-tagged dNrf2 (Figure S1J-N and pages 6-7). We find that although Nrf2 is present within embryonic MpTs, Nrf2 is cytoplasmic within MpTs during most of embryogenesis, only localising to the MpT nuclei by stage 17, and this nuclear Nrf2 localisation in MpTs continues through to adulthood (Figure S1J-N). The nuclear localisation of Nrf2 thus correlates well with the stages in which Nrf2 activity is observed within the MpTs.

4) Fig, 2H-L: The number of gH2Avd and cleaved caspase-3 positive cells and the number of hemocytes with corpses should be quantified.
We have now included this quantification in Figure 3I, L and O.

5) Fig. 3A-F: The abnormalities in larval and adult life may well be a consequence of the embryonic defects and thus may not reflect the role of Gadd45 at these later stages. This should be at least discussed.
This important point was raised by multiple reviewers and we have now have performed new experiments to uncouple the developmental and physiological roles of Gadd45 (as well as Nrf2) with temporally confined downregulations during post-embryonic and adult life (Figure 4T-Z and Figure S4U). To inhibit *gadd45* from larval life onwards we expressed *gadd45-RNAi* using the post-embryonic MpT driver *CapaR-gal4*; *CapaR-gal4* was further combined with a temperature sensitive Gal80 construct to inhibit *gadd45* also in adult MpTs only (discussed on pages 10-11). Our data suggest that Gadd45 is required both in embryonic and post-embryonic MpTs for stress resilience.

6) Page 9, end of last paragraph: Here, I would also cite Tsakiri et al., 2019, who showed negative consequences of Nrf2 overexpression in Drosophila. In particular, the authors should cite the paper by Suzuki et al., 2017 in this context (showing adverse effect of constitutive Nrf2 activation in the mouse kidney).
We apologise for missing these importance references, which we have now included in our Discussion on page 15.

7) Please mention the number of flies analyzed for each figure and supplementary figure in the

© 2021. Published by The Company of Biologists under the terms of the Creative Commons Attribution License (https://creativecommons.org/licenses/by/4.0/).

legends, even if no statistical analysis is presented (e.g. representative of ...).
We have now included more details in each Figure legend regarding the number of tubules or animals analysed in each experiment.

8) Some references that are in the reference list are not cited in the text and some references that are cited in the text are not in the reference list - please double check.
We apologise for these omissions and have now corrected the references.

Reviewer #2
1) The authors propose that actively transporting tubules are more metabolically active and therefore have higher oxidative stress – interesting idea, and makes sense. This case could be further bolstered by examining tubules during periods of decreased transport: wandering L3/pupae. (Hard to find published data on this, but anecdotally transport shuts down during these periods. Might be nice to get someone to do Ramsay assays on tubules from these stages to get the data published and correlate with oxidative stress.)
We thank the reviewer for this interesting suggestion, which we feel has greatly improved the paper (Figure 2, Figure S2 and pages 7-8). We have now analysed renal Nrf2 activity across all developmental stages (Figure 2A-B) and correlated this with renal tubule transport (via Ramsay assays, Figure 2C), mitochondrial activity (via JC-1 staining, Figure 2D) and oxidative stress (via a ratiometric ROS sensor, Figure 2E). As the Reviewer suggests, we find that renal Nrf2 activity is highest during periods when the tubules are most actively secreting (early L3 and adulthood) but is much reduced during periods when MpT transport is shut-down (wandering L3 and pupae) and this further correlates with levels of mitochondrial activity and mitochondrial ROS. Moreover, we have extended these analyses to compare Nrf2 activity, mitochondrial density, activity and redox status in principal versus stellate cells within adult tubules (Figure 2F-K).

2) Principal cell polyploidy is intriguing, as is decreased ploidy with loss of gadd45. Given the statement that “polyploidy may be a conserved mechanism for promoting stress resilience in vulnerable tissues,” is it possible to decrease ploidy through some other means, and examine effects on tubules? This would strengthen the argument for a role for ploidy in physiological stress resistance.
We have explored the effects of reducing MpT ploidy by tubule expression of *e2f-RNAi* (Figure 4J-N and Figure S4N-Q); although embryonic MpT morphogenesis is unaffected (Figure S4N-O), tubules with reduced ploidy exhibit increased DNA damage signalling (Figure 4K-M) and hosts have poorer survival than controls (Figure 4N) suggesting that increased ploidy plays a role in stress resilience in post-embryonic MpTs.

3) The manuscript shows related phenotypes for decreased and increased gadd45 and Nrf2. However, the relationship between gadd45 and Nrf2 is not examined. For example, does loss of gadd45 result in Nrf2 activation?
As described above in response to Reviewer #1 point #1, we have now explored the relationship between Nrf2 and Gadd45 within the renal tubules and analysed whether they perform overlapping functions (Figures 4-6). As the reviewer suggests, loss of *gadd45* does indeed result in Nrf2 activation (Figure 6E) which could reflect the increased inflammatory response in these animals driving more oxidative stress (please also see our response to Reviewer #1 point #1).

Minor comments.
1) Figure 1 B-E. Please state in figure legend what dotted white line is encircling.
We have now included more details of the structures labelled with the white dashed lines in the Figure legend.

2) Figure 1 H,H’. Please label stage. Looks like L3 but doesn’t say. Also, please indicate what part of L3 experiments were done in, given comment above about transport in early vs. wandering L3.
We have now labelled all developmental stages (being particularly careful to label eL3 vs wL3) on all Figure panels.

3) Figure 1 N-P. Please indicate in figure legend what arrowheads are pointing to. Looks like stellate cells but doesn’t say.
We have now indicated in the Figure legend that the arrowheads point to the stellate cell.

© 2021. Published by The Company of Biologists under the terms of the Creative Commons Attribution License (https://creativecommons.org/licenses/by/4.0/).

4) Figure 2 M-N. Would be nice to (optionally) show quantification of the Fucci experiments.
Along with our other additional quantification of the embryonic *gadd45-RNAi* phenotypes, we have included quantification of the Fucci (specifically *cyclin-B-RFP*) labelling In Figure 3R.

5) Figure 3 N, W and Figure S3 S. Are the control and gadd45/Nrf2 RNAi curves statistically different? The text says “tubule defects were associated with… significantly shorter lifespans than controls.” Similarly for Figure 4K, S.
We have now included the log-rank statistics for each survival curve and indicate which survival curves are statistically different (and those that are not) in Figures 4-6 and Figures S4-5.

6) Figure S3. In adults, byn-GAL4 is expressed in hindgut and not in Malpighian tubules. How late in development is byn-GAL4 expressed in the tubules?
***Byn-gal4* was employed as an earlier renal tubule Gal4 driver, as it drives Gal4-mediated expression in the embryonic hindgut/MpT primordium (Kispert et al., 1994, before *ctB-gal4* is active) and persists in embryonic MpT principal cells during MpT morphogenesis (Denholm et al., 2003); we have used *byn-Gal4* previously for GAL4-mediated transgene expression in the embryonic MpTs (Bunt et al., 2010). As the Reviewer suggests, although *byn-gal4* is a strong early embryonic MpT driver, *byn-gal4* is absent from larval or adult MpTs and for this reason we have now employed *CapaR-gal4* as an alternative post- embryonic MpT driver.**

Reviewer #3
1) Background knowledge on Gadd45 and Nrf2 should be included in the introduction part.
As also suggested by Reviewer 1, we have now included more detail about Nrf2 and Gadd45 within the Introduction (pages 3-4).

2) It is confusing that the authors mixed results and discussion part. To improve the readability of the paper, it would be better to separate these two parts.
We have now separated the Results and Discussion sections as the Reviewer suggests.

3) The authors acclaimed that Gadd45 and Nrf2 are required both in development and also during homeostasis. However, they failed to detangle developmental and the adult flies’ phenotype. Without this, it is difficult to know the real reason for the adult phenotype. For example, are the shortened lifespan and bloating abdomen in Gadd45 and Nrf2 knocking down fly due to the malformation of tubules during development or the malfunction of adult tubules? They should use Gal80TS lines to analyse the physiological of silencing Gadd45 and Nrf2 only in adults.
This important point was also raised by Reviewer #1 (point #5) and as described above, we have now have performed new experiments to uncouple the developmental and physiological roles of Gadd45 and Nrf2, with temporally confined downregulations during post-embryonic and adult life (Figure 4T-Z and Figure S4U). To inhibit *gadd45 or nrf2* from larval life onwards we expressed *RNAi* constructs using the post-embryonic MpT driver *CapaR-gal4*. *CapaR-gal4* was further combined with a temperature sensitive Gal80 construct to inhibit *gadd45* or *nrf2* only in adult MpTs (discussed on pages 10-11). Our data suggest that whilst Gadd45 is required both in embryonic and post-embryonic (larval and adult) MpTs (Figure 4), Nrf2 is required within both larval and adult MpTs (Figure 5) for stress resilience. Whilst the phenotypes obtained following inhibition of *nrf2* or *gadd45* only in adult MpTs were less severe than those obtained following earlier developmental knock-downs, our data nevertheless indicate both Nrf2 and Gadd45 play a role within adult MpTs for oxidative stress protection (for Nrf2, Figure O-Q) and host survival (Figure 4T-U and Figure 5L and 5N).

4) ctb-Gal4 is a good driver for embryogenic research; it is not the best driver to study adult phenotype. Using other principal cell drivers (uro-Gal4, or capaR-Gal4) combined with Gal80 system would answer both point 3 and point 4.
As the Reviewer suggests, we have employed *CapaR-gal4* in combination with Gal80^{ts} to analyse the role of Gadd45 and Nrf2 in adult MpTs.

5) It is difficult to link constitutive renal cryoprotection with Gadd45 based on current data. Current data only suggested Gadd45 is constitutively expressed throughout the fly’s life cycle. However, the rest data in Figure 1 and S1 only provided the evidence linking nrf2 with excessive ROS and stress challenge in the tubules. Does Gadd45 also respond to exogenous stress challenges like Nrf2? Does Gadd45 also protect tubules from oxidative stress like Nrf2?

© 2021. Published by The Company of Biologists under the terms of the Creative Commons Attribution License (https://creativecommons.org/licenses/by/4.0/).

We have now included additional data to show that *gadd45* is indeed induced in the renal tubules in response to exogenous stress (Figure 6C) and loss of *gadd45* causes a significant increase in oxidative damage (and Nrf2 activity) within post-embryonic renal tubules (Figure S4H) (please also see our response to Reviewer #1 point #1).

6) It seems like Nrf2 is required to control the excess ROS in the tubules. Can authors rescue the phenotype by reducing ROS? It would be great to know how Gadd45 and Nrf2 mediate their protective role.
We have included new data in Figure 5 that suggests expression of the Catalase enzyme reduces oxidative damage within the renal tubules and partially rescues the defects in adult survival of tubules lacking *nrf2* (Figure 5R-T). Conversely, we find that expression of Sod2 (superoxide dismutase) exacerbates the *nrf2-RNAi* phenotypes (Figure S5K-O) which is consistent with previous studies suggesting that superoxide dismutases can enhance H2O2 -mediated DNA damage (Midorikawa and Kawanishi, 2001).

We very much hope that the additional experiments we have performed and changes to the text we have now made to this revised version of our manuscript are satisfactory and that you and your referees are now happy to publish this study in *Development*.

Second decision letter

MS ID#: DEVELOP/2020/197343

MS TITLE: Metabolically active and polyploid renal tissues rely on graded cytoprotection to drive developmental and homeostatic stress resilience

AUTHORS: Katie Burbridge, Jack Holcombe, and Helen Weavers

I have now received all the referees reports on the above manuscript, and have reached a decision. The referees' comments are appended below, or you can access them online: please go to BenchPress and click on the 'Manuscripts with Decisions' queue in the Author Area.

The overall evaluation is very positive and we would like to publish a revised manuscript in Development, provided that the referees' comments can be satisfactorily addressed. As you will see, there are only a few outstanding comments from one reviewer, all of which concern the wording of the manuscript or the presentation of the data. Please attend to all of the reviewers' comments in your revised manuscript and detail them in your point-by-point response.

Reviewer 1

Advance summary and potential significance to field

The study provides important insight into the mechanisms underlying resilience of renal cells to oxidative and genotoxic stress and identifies Nrf2 and Gadd45 as key players.

Comments for the author

The authors have performed a large number of experiments to address the comments and criticisms of the reviewers. All my concerns and comments have been very well addressed. This is an important paper that will be of major interest to researchers in different fields.

© 2021. Published by The Company of Biologists under the terms of the Creative Commons Attribution License (https://creativecommons.org/licenses/by/4.0/).

Reviewer 2

Advance summary and potential significance to field

The manuscript has been substantially strengthened by extensive new data, which are informative and interesting – thank you for doing these experiments! In particular, in addition to the prior findings, the correlation between Nrf2 activity, tubule secretion, mitochondrial activity and mitrochondrial redox state across space and time has been strengthened; the role of polyploidy per se in tubule DNA damage and fly survival has been examined; the role of Nrf2 and gadd45 at different developmental stages has been better clarified; and rescue of loss of Nrf2 with catalase but not SOD overexpression has been demonstrated. The investigators have carefully and thoroughly addressed the reviewers' criticisms. I have only a few remaining minor points to be addressed.

Comments for the author

Minor comments:
1. In supplemental Figure 1, the label "cnc-GFP" is present in multiple panels. Non-fly people may not be aware that cnc is the Drosophila gene name for Nrf2 – this does not appear to be mentioned anywhere. The figure legend refers to "Nrf2-GFP" which may be the better label in the figure itself.
2. Results figure 2: the enrichment of Nrf2 activity in the main vs. initial segments also fits with the fact that while the main segment is secretory, the initial segment is not (Dow J Exp Biol 197: 421-428, 1994 and O'Donnell and Maddrell J Exp Biol 198: 1647-1653, 1995) – similar to the correlation of Nrf2 activity with tubule secretion at different developmental stages.
3. Supplemental Figure 3 legend, the first sentence seems to be missing a phrase after "MpT morphogenesis" ("is abnormal"?). Also, the legend states that * = $p<0.05$ and ** = $p<0.01$ in K and M, but there are no asterisks in these panels so unclear what this is referring to.
4. Figure 4 legend: "DNA damage… and tubule morphology… also disturbed" – grammar needs to be fixed.
5. Supplemental Figure 5J: figure legend states that "expression of catalase partially rescues the bloating phenotype…" but statistics in the figure state "ns" for the difference between nrf2[RNAi] and nrf2[RNAi] +
UAS-catalase.

Reviewer 3

Advance summary and potential significance to field

Describe the ability of Drosophila Malpiguian tubules to resist stress including ROS stresses

Comments for the author

The paper has been improved and is now suitable for publications

---

Second revision

Author response to reviewers' comments

"Metabolically active and polyploid renal tissues rely on graded cytoprotection to drive developmental and homeostatic stress resilience"

We are grateful for the additional suggestions from Reviewer 2, which we feel have further improved the paper; we have now addressed these concerns with a combination of text and Figure changes. The text below provides a detailed response to individual reviewer comments with our response to each suggestion/comment highlighted in bold following the text of the

© 2021. Published by The Company of Biologists under the terms of the Creative Commons Attribution License (https://creativecommons.org/licenses/by/4.0/).

reviews. We have also highlighted each individual revision within the revised manuscript in yellow.

Reviewer #2:
1) In supplemental Figure 1, the label “cnc-GFP” is present in multiple panels. Non-fly people may not be aware that cnc is the Drosophila gene name for Nrf2 – this does not appear to be mentioned anywhere. The figure legend refers to “Nrf2-GFP” which may be the better label in the figure itself.
We apologise for the confusion here. We have now introduced that the *Drosophila* homolog of Nrf2 is termed *cnc* in the main text (page 6) and explained in the legend to Figure S1 that we have used a GFP-tagged construct of *Drosophila cnc* to analyse Nrf2 localisation. We would prefer to use ‘cnc-GFP’ in the Figure to ensure consistency with the nomenclature used for this specific transgenic construct in stock centre records.

2) Results figure 2: the enrichment of Nrf2 activity in the main vs. initial segments also fits with the fact that while the main segment is secretory, the initial segment is not (Dow J Exp Biol 197: 421- 428, 1994 and O’Donnell and Maddrell J Exp Biol 198: 1647-1653, 1995) – similar to the correlation of Nrf2 activity with tubule secretion at different developmental stages.
We very much appreciate this suggestion and have now mentioned this interesting correlation in the main text on page 8 and included these additional references.

3) Supplemental Figure 3 legend, the first sentence seems to be missing a phrase after “MpT morphogenesis” (“is abnormal”?). Also, the legend states that * = $p<0.05$ and ** = $p<0.01$ in K and M, but there are no asterisks in these panels so unclear what this is referring to.
We have now modified the legend for Figure S3 after “MpT morphogenesis” and also corrected the reference to the missing asterisks.

4) Figure 4 legend: “DNA damage… and tubule morphology… also disturbed” – grammar needs to be fixed.
We apologise for this grammatical error and have now modified the text appropriately.

5) Supplemental Figure 5J: figure legend states that “expression of catalase partially rescues the bloating phenotype…” but statistics in the figure state “ns” for the difference between nrf2[RNAi] and nrf2[RNAi] + UAS-catalase.
We apologise for the confusion here and have now modified the text in the legend of Figure S5.

Third decision letter

MS ID#: DEVELOP/2020/197343

MS TITLE: Metabolically active and polyploid renal tissues rely on graded cytoprotection to drive developmental and homeostatic stress resilience

AUTHORS: Katie Burbridge, Jack Holcombe, and Helen Weavers
ARTICLE TYPE: Research Article

I am happy to tell you that your manuscript has been accepted for publication in Development, pending our standard ethics checks.

© 2021. Published by The Company of Biologists under the terms of the Creative Commons Attribution License (https://creativecommons.org/licenses/by/4.0/).
